# Optical nanomaterial-based detection of biomarkers in liquid biopsy

**DOI:** 10.1186/s13045-024-01531-y

**Published:** 2024-03-14

**Authors:** Young Jun Kim, Won-Yeop Rho, Seung-min Park, Bong-Hyun Jun

**Affiliations:** 1 Department of Bioscience and Biotechnology, Konkuk University, Seoul, 05029 Republic of Korea; 2 School of International Engineering and Science, Jeonbuk National University, Chonju, 54896 Republic of Korea; 3 School of Chemistry, Chemical Engineering and Biotechnology, Nanyang Technological University, Singapore, 637459 Singapore

**Keywords:** Liquid biopsy, Optical nanoparticles, Circulating tumor markers, Circulating tumor cells, Circulating exosomes, Circulating tumor DNAs

## Abstract

Liquid biopsy, which is a minimally invasive procedure as an alternative to tissue biopsy, has been introduced as a new diagnostic/prognostic measure. By screening disease-related markers from the blood or other biofluids, it promises early diagnosis, timely prognostication, and effective treatment of the diseases. However, there will be a long way until its realization due to its conceptual and practical challenges. The biomarkers detected by liquid biopsy, such as circulating tumor cell (CTC) and circulating tumor DNA (ctDNA), are extraordinarily rare and often obscured by an abundance of normal cellular components, necessitating ultra-sensitive and accurate detection methods for the advancement of liquid biopsy techniques. Optical biosensors based on nanomaterials open an important opportunity in liquid biopsy because of their enhanced sensing performance with simple and practical properties. In this review article, we summarized recent innovations in optical nanomaterials to demonstrate the sensitive detection of protein, peptide, ctDNA, miRNA, exosome, and CTCs. Each study prepares the optical nanomaterials with a tailored design to enhance the sensing performance and to meet the requirements of each biomarker. The unique optical characteristics of metallic nanoparticles (NPs), quantum dots, upconversion NPs, silica NPs, polymeric NPs, and carbon nanomaterials are exploited for sensitive detection mechanisms. These recent advances in liquid biopsy using optical nanomaterials give us an opportunity to overcome challenging issues and provide a resource for understanding the unknown characteristics of the biomarkers as well as the mechanism of the disease.

**Graphical abstract:**

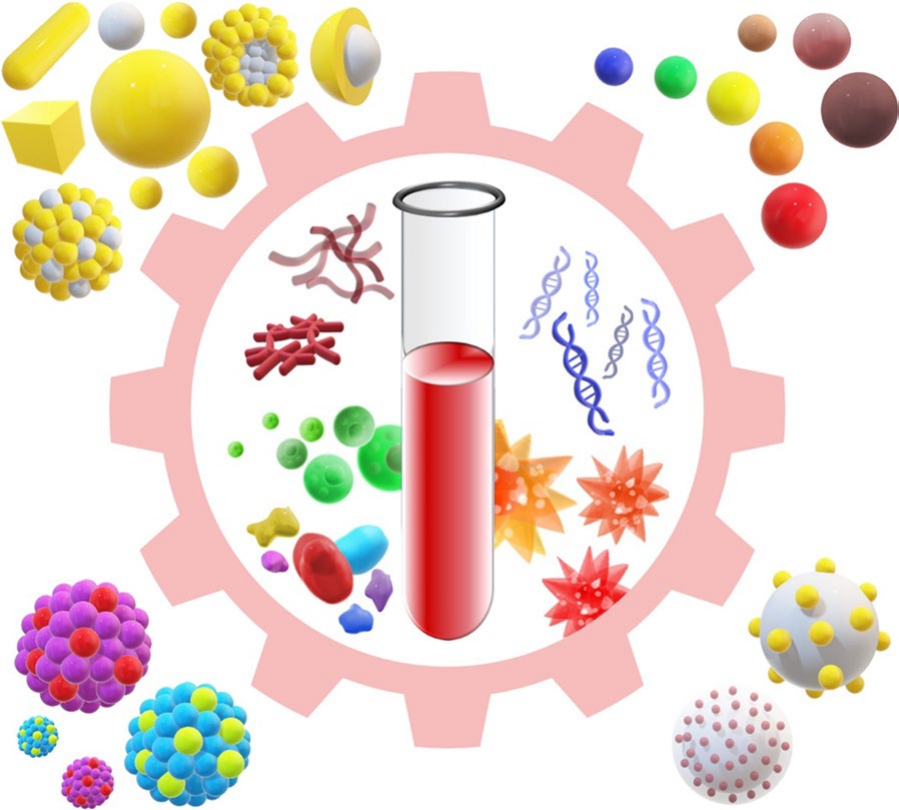

## Introduction

Over the past decade, liquid biopsy has emerged as a compelling alternative to the standard tissue biopsy used for cancer diagnosis [1, 2]. Traditional tissue biopsy, which involves surgically extracting a piece of tumorous tissue, provides physicians with direct information about a patient's lesion. However, this procedure can be risky, painful, and burdensome, making frequent monitoring through routine or repetitive examinations impractical [3]. Furthermore, certain lesions may be inaccessible for tissue biopsy due to their location or size, posing a significant obstacle to early diagnosis. It is also important to note that a tissue biopsy may not fully capture the complex profile of the primary tumor [4]. The increasing understanding of intratumor heterogeneity indicates that analyzing a specific segment of a lesion may yield only limited information about a localized area [5]. Therefore, the need for alternatives like liquid biopsy, which can provide a more comprehensive view of the tumor, is clear.

On the other hand, liquid biopsy takes the biomarkers that are shed into the bloodstream or other biofluids like saliva, urine, sweat, and interstitial fluid [6, 7]. This minimally invasive approach does not require risky, painful, and burdensome procedures, permitting the possibility of regular disease monitoring through routine analysis of biofluids (e.g., blood tests or other fluid sample tests). This broader range of potential samples helps to ensure a comprehensive understanding of the disease progression. Regarding tumor heterogeneity, liquid biopsy provides insights that are not confined to a specific portion of the lesion. Given the hypothesis that biomarkers in the bloodstream correlate with metastatic processes, liquid biopsies could be instrumental in deciphering the intricate profile of primary tumors [8].

While liquid biopsy offers numerous advantages, it cannot, at the present stage, replace traditional diagnostic procedures, including tissue biopsy [9]. One fundamental challenge of liquid biopsy lies in its conceptual intricacy and the detection of its key biomarkers. The primary biomarkers in liquid biopsy, circulating tumor cells (CTCs), circulating tumor DNAs (ctDNAs), and tumor-derived exosomes, are notably low in abundance or purity. They exist as minute fractions amidst other blood cells, cell-free DNAs, and normal extracellular vesicles and are widely distributed in large-volume biofluids. As such, both efficient enrichment and ultra-sensitive detection are paramount for the future development of liquid biopsy techniques [10, 11]. In the quest to integrate liquid biopsy into clinical practice, this review article specifically emphasizes optical biosensors rooted in advancements in optical nanomaterials. Optical detections are advantageous in sensitivity, stability, and immunity to external disturbance, thus achieving a high signal-to-noise ratio with a relatively simple procedure [12]. Considering the complex environment of biofluids, optical detection can be an ideal candidate for liquid biopsy. Furthermore, their detection performance can simply be enhanced by the innovation of nanomaterial-based probes. Nanomaterials, which exhibit substantially increased surface-area-to-volume ratio, can be a support for other indicators (e.g., organic dye) or be an indicator itself. The unique features that are different from their bulk corresponding materials also enable us to employ versatile detection strategies with the enhanced efficiency of the chemical and catalytic reactions [13]. Optical nanomaterials, including metallic nanoparticles (NPs), metallic oxide NPs, quantum dots (QDs), upconversion nanoparticles (UCNPs), and carbon nanomaterials are able to act as a sensitive optical nanoprobe solely or cooperatively with their own characteristics. In the enrichment of CTC or other ctDNA fragments, nanomaterials have already been employed for effective enrichment, potentially allowing them to serve dual functions—enrichment and detection [14, 15]. In addition, the superiority of optical nanomaterials lies in their unique characteristics that enable precise sensing mechanisms, providing a pathway to achieve both ultra-sensitive detection and accurate quantification. Recently, cutting-edge optical technology has been employed to detect glucose levels in vivo using wearable devices, underscoring the increasing clinical practicality of these methods. Hence, the choice of nanomaterials and the design of the nanoprobes are crucial factors in enhancing the resulting sensing performance.

## Liquid biopsy and current routine diagnostics

As we mentioned above, the nomenclature of liquid biopsy originated from tissue (solid) biopsy, meaning an alternative concept. Unlike traditional biopsy, which uses needles to cut and collect the tissues, sometimes assisted by aspirators or vacuum devices, liquid biopsy will be based on routine diagnostic procedures for blood testing and urinalysis today. It is the primary advantage of liquid biopsy. Historically, biofluids like blood and urine have always been described as a snapshot of health conditions because they reflect the metabolism, organ function, and body balance. Blood testing monitors cells, proteins, enzymes, hormones, and other chemical substances in blood to evaluate the function of the body through complete blood count tests, metabolic tests, electrolyte tests, and so on [16]. It is also helpful in finding evidence of disorders and diseases, such as allergies, diabetes, blood clotting disorders, autoimmune diseases, endocrine system disorders, cancer, heart disease, and infectious diseases. Liquid biopsy conducted this process by collecting the disease-related or disease-derived biomarkers, such as ctDNAs, exosomes, and CTCs. Assuming that these biomarkers reflect the molecular and genomic characteristics of parental cells, they replace the tissues of primary tumors. For example, ctDNAs share the same genetic defects as their origin tumor DNAs. In addition, liquid biopsy is advantageous in relevance of information. The tissues obtained by the traditional biopsy often fail to represent the complex characteristics of the tumor due to the heterogeneity of the tumor. However, the biomarkers collected via liquid biopsy carry information that is not localized in specific tissue-taking spots. Further, these short-lived biomarkers may contain recently generated information about the current status of disease. In spite of these potentials, both heterogeneity and short half-life of biomarkers, along with rarity, are problematic in developing accurate detection methods.

Next, we need to consider the pre- and post-procedures of biopsy. Traditional biopsy is inseparable from medical imaging, such as X-ray imaging, ultrasound imaging, computational tomography (CT), positron emission tomography (PET), Single-photon emission computed tomography (SPECT), and magnetic resonance imaging (MRI). These techniques visualize the structures and functions in the body; thus, they have a crucial role in the diagnostic procedures by figuring out the injury and illness [17]. For these reasons, imaging is the basis of decision-making for performing a biopsy by confirming the site of abnormality and also giving guidance during the biopsy procedure. It should be pointed out that optical biosensors were originally developed with the idea of replacing medical imaging systems, as affordable and accessible options. In this context, the analysis of LB biomarkers via optical detection is analogous to the relationship between traditional biopsy and medical imaging.

## Optical nanomaterial-based detection of LB biomarkers

Although there have been important technical milestones over the last decades, the remaining challenges for liquid biopsy are substantial. The major problem here is a lack of accuracy. Considering that the concept of liquid biopsy presupposes the detection of low-abundant analytes from the large-volume biofluid, the urgent requirement for liquid biopsy would be ultrasensitive detection and/or highly efficient enrichment [18]. In this context, optical biosensing can be an ideal candidate for the realization of liquid biopsy. First, it provides a relatively simple and straightforward recognition of the analytes of interest. Second, enhancement of the sensing performance can be achieved by the design and combination of optical nanomaterials. Third, it is suitable for the measurement in complex samples like biofluid due to less interference from the background. These advantages can be key factors in achieving the current assignment of the liquid biopsy.

**Fig. 1:**
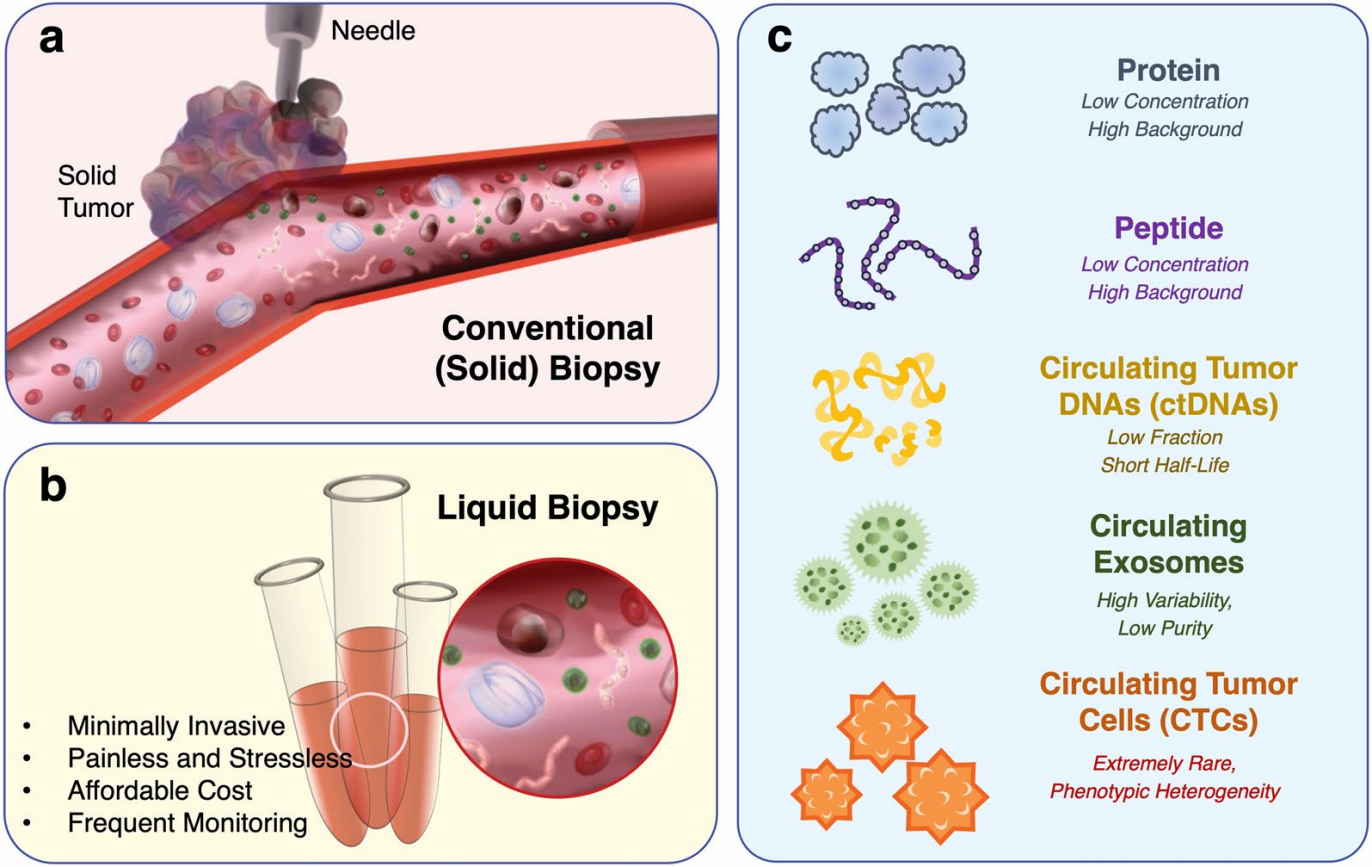
Biopsy and LB biomarkers: **a** the concept of conventional (solid) biopsy; **b** the concept of liquid biopsy and its advantages. **c** LB biomarkers and their own challenges

In this section, we summarized the recent studies that achieved improved sensing performance for LB biomarkers with the help of optical nanomaterials. The subsections are categorized by proteins, peptides, ctDNAs, miRNAs, exosomes, and CTCs, focusing on the specific issues of each marker. In this review, we categorized the biomarkers for liquid biopsy (“LB biomarkers”), including both traditional and revolutionary ones, into five groups: proteins, peptides, ctDNAs and miRNAs (nucleic acids), exosomes (extracellular vesicles), and circulating tumor cells (cells). Figure 1 illustrates the concept of liquid biopsy and the categorization of LB biomarkers and their challenging issues. Tables 1 and 2 describe the ranges of the concentration of LB biomarkers in biofluid. In Table 1, the concentration of each LB biomarker follows the cut-off value when it is currently utilized in clinical blood tests or urinalysis (e.g., traditional protein LB biomarkers and some peptide LB biomarkers). In Table 2, the expected concentration range refers to the previous reports and studies in the case of newly emerging LB biomarkers. These study results for newly emerging LB biomarker studies need to be interpreted with caution because there has not been a clear reference range, and the extensive investigation is still ongoing.

**Table 1:** Concentration of LB biomarkers in biofluid based on reference cut-off value in clinical blood tests

Classification	Biomarker	Disease	Biofluid	Concentration range		Note
				Normal	Abnormal	
Protein	Alphafetoprotein (AFP)	Liver Cancer	Blood/Serum	≤ 20 ng/mL	> 400 ng/mL	Blood Tests [19]
Protein	Bladder Tumor Antigen (BTA)	Bladder Cancer	Urine	≤ 14 U/mL	> 14 U/mL	Blood Tests [20]
Protein	Cancer Antigen 125 (CA 125)	Ovarian Cancer	Blood/Serum	≤ 35 U/mL	> 35 U/mL	Blood Tests [21]
Protein	Cancer Antigen 19–9 (CA 19–9)	Pancreatic Cancer	Blood/Serum	≤ 37 U/mL	> 37 U/mL (360 pM)	Blood Tests [22]
Protein	Carcinoembryonic Antigen (CEA)	Colorectal Cancer Lung Cancer	Blood/Serum	≤ 5 ng/mL	> 5 ng/mL	Blood Tests [23]
Protein	Cytokeratin Fragment (CYFRA-21–1)	Lung Cancer	Blood/Serum	≤ 3.3 ng/mL	> 3.3 ng/mL	Blood Tests [24]
Protein	Nuclear Matrix Protein 22 (NMP22)	Bladder Cancer	Urine	≤ 14 U/mL	> 14 U/mL	Blood Tests [25]
Protein	Prostate Cancer Antigen (PSA)	Prostate Cancer	Blood/Serum	≤ 4 ng/mL	> 10 ng/mL	Blood Tests [26]
Protein	Neuron-Specific Enolase (NSE)	Lung Cancer	Blood/Serum	–	347 pM (16.3 ng/mL)	Blood Tests [27]
Protein	Hepatitis B Surface Antigen (HBsAg)	Hepatitis B and Hepatocellular Carcinoma	Blood	≤ 0.05 U/mL	> 0.05 IU/mL	Blood Test [28]
Protein	Hepatitis C Core Antigen (HCcAg)	Hepatitis C	Blood	≤ 0.06 pg/mL	> 0.06 pg/mL	Blood Test [29]
Peptide	Brain Natriuretic Peptide (BNP)	Heart Failure	Plasma	≤ 100 pg/mL	> 100 pg/mL	Blood Test [30]
Peptide	N-terminal proBNP (NTproBNP)	Heart Failure	Plasma	≤ 300 pg/mL	> 300 pg/mL

**Table 2:** Concentration of LB biomarkers in biofluid based on the study with clinical samples

Classification	Biomarker	Disease	Biofluid	Concentration range		Note
				Healthy control	Patients	
Protein	T-Tau	Alzheimer's Disease	CSF	507 ± 254 pg/mL	828 ± 375 pg/mL	Study [31] (*n* = 54)
			Plasma	4.43 ± 2.83 pg/mL	8.80 ± 10.1 pg/mL	
Protein	P-Tau	Alzheimer's Disease	CSF	73.4 ± 20.5 pg/mL	123 ± 49.2 pg/mL	Study [31] (*n* = 54)
Protein	P-Tau 181	Alzheimer's Disease	CSF	15.7 ± 13.5 pg/mL	108.5 ± 99.6 pg/mL	Study [32] (*n* = 21)
			Plasma	1.91 ± 1.06 pg/mL	3.6 ± 1.8 pg/mL	
			Plasma	1.5 ± 1.1 pg/mL	4.7 ± 2.0 pg/mL	Study [33] (*n* = 38)
Protein	P-Tau 231	Alzheimer's Disease	CSF	30.1 ± 36.1 pg/mL	262.0 ± 230.1 pg/mL	Study [32] (n = 21)
			Plasma	2.1 ± 1.2 pg/mL	5.4 ± 2.0 pg/mL	
Peptide	Beta-Amyloid 40 (Aβ40)	Alzheimer's Disease	CSF	5.3–11.8 ng/mL	4.7–23.4 ng/mL	Study [34] (*n* = 36)
			CSF	4.7 ± 1.7 ng/mL	4.4 ± 1.8 ng/mL	Study [35] (*n* = 57)
			Plasma	35–490 pg/mL	100–770 pg/mL	Study [34] (*n* = 78)
			Plasma	276.7 ± 66.1 pg/mL	244.3 ± 105.8 pg/mL	Study [35] (*n* = 57)
			Plasma	288.0 pg/mL	272.4 pg/mL	Study [36] (*n* = 18)
			Plasma	–	150–300 pg/mL (33–67 pM)	Study [37]
Peptide	Beta-Amyloid 42 (Aβ42)	Alzheimer's Disease	CSF	25–250 pg/mL	25–325 pg/mL	Study [34] (*n* = 36)
			CSF	554.0 ± 195.0 pg/mL	289.5 ± 103.8 pg/mL	Study [35] (*n* = 57)
			Plasma	25–905 pg/mL	25–880 pg/mL	Study [34] (*n* = 78)
			Plasma	19.6 ± 5.2 pg/mL	13.2 ± 7.3 pg/mL	Study [35] (*n* = 57)
			Plasma	37.1 pg/mL	30.1 pg/mL	Study [36] (*n* = 18)
			Plasma	–	5–30 pg/mL (1–7 pM)	Study [37]
Nucleic Acid	Circulating Free DNA (cfDNA) or Circulating Tumor DNA (ctDNA)	11 Different Types of Cancer *	Serum	13 ± 3 ng/mL (0–100 ng/mL)	180 ± 38 ng/mL (0—5000 ng/mL)	Study [38] (*n* = 173)
		Lung Cancer	Serum	0–30 ng/mL	0—1000 ng/mL	Study [39]
		Prostate Cancer	Plasma	7.9 ± 4.0 ng/mL (0.29–16.9 ng/mL)	13.8 ± 28.1 ng/mL ** (1–1380 ng/mL)	Study [40] (*n* = 122)
		Breast Cancer	Plasma	9 ng/mL (1.2–41 ng/mL)	32.4 ng/mL ** (2.83–6820 ng/mL)	Study [41] (*n* = 111)
Extracellular Vesicle	Exosome	–	Plasma	0.88 × 10^8^–13.38 × 10^8^ exosomes/mL	–	Study [42]
Cell	Circulating Tumor Cell (CTC)	Prostate Cancer	Whole Blood	**–**	75 ± 333 cells / 7.5 mL	Study [43] (*n* = 123)
		Breast Cancer	Whole Blood	–	84 ± 885 cells / 7.5 mL	Study [43] (*n* = 422)
		Colorectal Cancer	Whole Blood	–	4 ± 11 cells / 7.5 mL	Study [43] (*n* = 196)
		Lung Cancer	Whole Blood	–	30 ± 178 cells / 7.5 mL	Study [43] (*n* = 99)
					0–7 cells / 2.0 mL	Study [15] (*n* = 11)
		Ovarian Cancer	Whole Blood	–	6 ± 16 cells / 7.5 mL	Study [43] (*n* = 29)
		Gastric Cancer	Whole Blood	–	24 ± 83 cells / 7.5 mL	Study [43] (*n* = 9)
		Bladder Cancer	Whole Blood	–	42 ± 107 cells / 7.5 mL	Study [43] (*n* = 7)
		Pancreatic Cancer	Whole Blood	–	2 ± 6 cells / 7.5 mL	Study [43] (*n* = 16)

^*^Lymphoma, lung, ovary, uterus, cervical, glioma, head-neck, central nervous system, breast, colon, and rectal tumors. **Metastatic case

The representative examples of optical nanomaterials utilized in liquid biopsy are metallic NPs (e.g., Au, Ag, Pt), bimetallic NPs, metallic oxide NPs, QDs, UCNPs, carbon nanodots (CNDs), carbon quantum dots (CQDs), graphene quantum dots (GQDs). Other nanomaterials, including graphene, graphene oxide (GO), single-walled carbon nanotubes (SWCNTs), multi-walled carbon nanotubes (MWCNTs), metal–organic frameworks (MOFs), MXenes, silica NPs, polymeric NPs, and magnetic NPs, were utilized for the development of efficient sensing mechanisms. Each has unique characteristics that can be utilized in sensing mechanisms and thus measured by various compatible detection methods, such as colorimetric detection, fluorescence detection, chemiluminescence detection, electrochemiluminescence (ECL) detection, surface plasmon resonance (SPR) spectroscopy, localized surface plasmon resonance (LSPR) sensing, surface-enhanced Raman scattering (SERS) spectroscopy, CD Spectrometry, upconversion-linked immunosorbent assays (ULISA), X-ray fluorescence spectrometry, laser desorption ionization mass Spectrometry (LDI-MS), and inductively coupled plasma mass spectroscopy (ICP-MS). Although there are differences in sensitivity among these techniques, the direct comparison among studies is somewhat difficult. The differences in sensing performance of biosensors can be made from different experimental settings, experimental procedures, and various factors, including assay format, affinity of biorecognition molecules (e.g., antibody and aptamer), type of nanomaterials, types of indicators, sample matrix, and sample volume.

### Protein

As a traditional biomarker, protein in the biofluid has long been utilized to monitor individuals’ health status. For now, this procedure, which is often described as a “blood test,” is included in part of routine medical check-ups [44]. The representative examples are alpha-fetoprotein (AFP) for liver cancer, carcinoembryonic antigen (CEA) for colorectal cancer, carbohydrate antigen 19-9 (CA 19-9) for pancreatic cancer, cancer antigen 125 (CA 125) for ovarian cancer, and prostate-specific antigen (PSA) for prostate cancer. Because these protein markers usually have an established reference range to discriminate the normal and abnormal concentration, the required sensing performance is relatively obvious. For example, the clinical cut-off range of the CEA marker is around 5 ng/mL for both lung and colorectal cancers [45]. Commercial ELISA kits can detect down to 0.2 ng/mL, and most biosensing studies report more sensitive LODs than ELISA [46–48]. Likewise, PSA, the most common screening target in prostate cancer tests, is usually under 4 ng/mL in the blood obtained from healthy individuals (Table 1). If it is elevated to above borderline (> 10 ng/ml), the possibility of having prostate cancer reaches around 50% [26]. However, the background level and borderline can be shifted by age and other health conditions. The sensitivity of the commercial ELISA kits is around 8 pg/mL, and the recently suggested biosensing studies claimed an impressive performance over ELISA with sub-picomolar detection [49, 50]. However, these traditional biomarkers have limitations in the criteria of liquid biopsy. First, these markers are naturally present in the blood at a certain level, regardless of disease or other health issues. Second, protein LB makers do not provide a holistic view of the disease because they usually designate one or two specific cancers. For instance, a PSA level in the normal range tells us the patients might not have prostate cancer, but it does not mean they do not have any type of cancer. Furthermore, some cancers do not have analogous protein biomarkers. Thus, there is a limitation that the detection of protein level cannot offer comprehensive information on the health status of the patients, and separate tests may be required for each marker. Third, the background level of these protein markers in the blood is usually not low and highly varied according to individual differences, such as age, sex, race, and other factors. Fourth, these biomarkers are not able to contribute to the significant promises of liquid biopsy, including prediction of prognosis and therapeutic responses. The protein level itself is not relevant to the understanding of tumor heterogeneity. For this reason, there has been a question over its classification: do we need to include these traditional protein markers as a part of the novel concept of liquid biopsy? Some researchers agree, but some disagree [51, 52]. Nevertheless, protein LB biomarkers also face a turning point in the liquid biopsy era thanks to the advances in technologies. In spite of the fact that protein detection is far from an alternative concept to tissue biopsy, proteins also carry out information derived from disease-related cells and are able to support clinal implementation [52].

Traditional biomarkers like protein have been a target analyte since the first page of the biosensor history. To evaluate the risk of diseases at the early stage, the primary goal of biosensors is ultrasensitive and quantitative detection. The representative optical nanomaterial-based detections of protein LB markers are described in Table 3. The studies conducted multiplexed detection are provided in Table 4.

**Table 3:** The optical nanomaterial-based biosensors for the detection of protein LB markers

Biomarker	Disease	Optical nanomaterial	Biorecognition element	Detection method	Matrix	Limit of detection	Linear range	Clinical sample [a]	Note
Alpha-fetoprotein (AFP)	Liver Cancer	AuNPs	Antibody	Localized Surface Plasmon Resonance (LSPR)	Buffer Serum	0.1 ng/mL 2.33 ng/mL	0.1 ng/mL–100 ng/mL 2.33 ng/mL–143.74 ng/mL	–	2009 [53]
	Liver Cancer	AuNPs (+ Magnetic NPs)	Antibody	Chemiluminescence Detection	Buffer	5 pg/mL	0.008—0.3 ng/mL	–	2009 [54]
	Liver Cancer	AgNPs	Aptamer	Surface Enhanced Raman Scattering (SERS) Spectroscopy	N/A	0.097 aM	0.2–20 aM	Serum (*n* = 10)	2015 [55]
	Liver Cancer	Au Nanomashroom	Antibody	LSPR	Buffer	24 ng/mL	20–200 ng/mL	Serum (*n* = 3)	2015 [56]
	Liver Cancer	Ag@SiO_2_ NPs	Antibody	SERS Spectroscopy	Buffer Blood	3.0 ng/mL 17.0 ng/mL	20–300 ng/mL 50–500 ng/mL	–	2017 [57]
	Liver Cancer	AuNPs	Antibody	LSPR	N/A	150 ng/mL	1 ng/mL–1 ug/mL	–	2017 [58]
	Liver Cancer	Au@AgNPs	Antibody	LSPR	N/A	3.3. fg/mL	10^−12^–10^−8^ g/mL	Serum (*n* = 15)	2020 [59]
	Liver Cancer	Au Nanobipyramid	Antibody	SERS Spectroscopy	serum	0.085 pg/mL	3–10 pg/mL		2023 [60]
Carcinoembryonic Antigen (CEA)	Lung Cancer	Au Nanoflower	Antibody	SERS Spectroscopy	N/A	0.01 fg/mL	0.01 fg/mL–1 ng/mL	–	2014 [46]
	Lung Cancer	Au@SiO^2^ Nanorods	Antibody	SERS Spectroscopy	Buffer	0.86 fg/mL	1 fg/mL–10 ng/mL	–	2014 [47]
	Lung Cancer	AuNPs	Antibody	Surface Plasmon Resonance (SPR) Spectroscopy	Buffer	1.0 ng/mL	1–60 ng/mL	–	2015 [61]
	Lung Cancer	UCNPs (NaYF_4_:Yb,Er)	Aptamer	Fluorescence Detection	Buffer	7.9 pg/mL	0.03–6 ng/mL	Serum (*n* = 5)	2019 [48]
					Serum	10.7 pg/mL	0.03–6 ng/mL		
	Lung Cancer	MoS_2_@AuNPs and Fe_3_O_4_@AuNPs	Antibody	SERS Spectroscopy	N/A	0.033 pg/mL	0.0001–100.0 ng/mL	–	2020 [62]
Cancer Antigen 125 (CA 125)	Ovarian Cancer	CdTe QDs	Antibody	Electrochemiluminescence (ECL) Detection	Buffer	0.0012 U/mL	0.005–50 U/mL	–	2013 [63]
	Ovarian Cancer	Graphene QDs	Antibody	Chemiluminescence Detection	Buffer	0.05 U/mL	0.1–600 U/mL	–	2014 [64]
	Ovarian Cancer	AuNPs	Antibody	Colorimetric Detection	Buffer	30 U/mL	0–1000 U/mL	–	2017 [65]
	Ovarian Cancer	AgNPs and UCNPs (NaYF_4_:Yb,Tm)	Antibody	Fluorescence Detection	Buffer	120 pg/mL	5–100 ng/mL	–	2019 [66]
	Ovarian Cancer	Carbon QDs	N/A	Fluorescence Resonance Energy Transfer (FRET)	Buffer	0.66 U/mL	0.01–129 U/mL	Serum (*n* = 12)	2021 [67]
	Ovarian Cancer	Graphitic Carbon Nitride and SiO_2_@CdTe/CdS QDs	Antibody	Electrochemiluminescence resonance energy transfer (ECL-RET)	Buffer	0.034 mU/mL	0.0001–10 U/mL	–	2021 [68]
Cancer Antigen 19-9 (CA 19-9)	Pancreatic Cancer	Ag@SiO_2_@Ag Core–Shell NPs	Antibody	SERS Spectroscopy	N/A	0.5 U/mL	0.5–1000 U/mL	–	2016 [69]
	Pancreatic Cancer	SiO_2_ NPs and AgNPs	Antibody	SERS Spectroscopy	N/A	1.3 × 10^−3^ U/mL	10^–1^–10^3^ IU/mL	–	2016 [70]
	Pancreatic Cancer	Ag@PSPAA@Ag Core–Shell Nanomushroom	Antibody	SERS Spectroscopy	Buffer	10^–4^ U/mL	0.0001–10 U/mL	–	2021 [71]
	Pancreatic Cancer	SiO_2_-coated Gd-doped UCNPs (NaYF_4_:Yb^3+^, Er^3+^)	Antibody	Upconversion-Linked Immunosorbent Assays (ULISA)	N/A	5 U/mL	5–20,000 U/mL	–	2021 [72]
	Pancreatic Cancer	Au Nanoflowers and Red Phosphorus Nanoplates	Antibody	SERS Spectroscopy	Buffer	7.41 × 10^–5^ IU/mL	10^–4^–10^2^ IU/mL	Serum (*n* = 5)	2022 [73]
Cancer Antigen 15-3 (CA 15-3)	Breast Cancer	CdS QDs	Antibody	Fluorescence Detection	N/A	0.002 U/mL	N/A	–	2017 [74]
	Breast Cancer	AuNPs	Antibody	FRET	N/A	0.9 × 10^–6^ U/mL	1.0 × 10^−6^–5.0 × 10^−3^ U/mL	–	2018 [75]
	Breast Cancer	Au–Ag@zein	Antibody	ECL Detection	Buffer	0.0003 U/mL	0.001–100 U/mL	–	2023 [76]
Prostate Cancer Antigen (PSA)	Prostate Cancer	SiO_2_@Ag@SiO_2_ NPs	Antibody	SERS Spectroscopy	Buffer	0.11 pg/mL	0.001–1000 ng/mL	–	2016 [49]
	Prostate Cancer	Ag@SiO_2_@SiO_2_-RuBpy	Antibody	Metal-Enhanced Fluorescence (MEF) Detection	Buffer Diluted Serum	27 pg/mL 31 pg/mL	0.1 ng/mL – 100 ng/mL	–	2017 [77]
	Prostate Cancer	UCNPs (NaYF_4_:Yb^3+^,Er^3+^) and Au NPs	Antibody	Luminescence Resonance Energy Transfer (LRET)	Serum	1.0 pM	0–500 pM	–	2018 [78]
	Prostate Cancer	UCNPs (NaYF_4_:Yb^3+^,Er^3+^)	Antibody	ULISA	Buffer	23 fg/mL	0.1–100 pg/mL	–	2019 [50]
		UCNPs (NaYF_4_:Yb^3+^,Tm^3+^)				24 fg/mL	1–100 pg/mL		
	Prostate Cancer	Au Nanodisk Array	Antibody	Fiber-optic LSPR	Buffer	0.1 pg/mL	0.1 pg/mL–1.0 ng/mL	–	2019 [79]
	Prostate Cancer	ZnGeO:Mo NRs and Au@Ag@SiO_2_ NPs	Aptamer	Luminescence Detection	Buffer	9.2 pg/mL	10 pg/mL–10 ng/mL	–	2019 [80]
	Prostate Cancer	CdTe@SiO_2_ NPs	Antibody	Fluorescence Detection	N/A	0.003 ng/mL	0.01–5 ng/mL	–	2019 [81]
	Prostate Cancer	AuNPs	Antibody	Colorimetric Detection	N/A	0.23 ng/mL	0.25–2500 ng/mL	–	2020 [82]
	Prostate Cancer	SiO_2_@Au@AgNPs	Antibody	SERS Spectroscopy	N/A	0.006 ng/mL	N/A	–	2021 [83]
	Prostate Cancer	AgNPs and Si Nanowire	Aptamer	SERS Spectroscopy	Buffer	0.1 μg/mL	0.1–20 μg/mL	–	2021 [84]
	Prostate Cancer	SiO_2_@Ag@SiO_2_ NPs	Antibody	Lateral Flow Assay (LFA)	N/A	1.1 ng/mL	N/A	Serum (*n* = 7)	2021 [85]
	Prostate Cancer	SiO_2_@Au–Ag NPs	Antibody	LFA	N/A	0.30 ng/mL	0.3–10,0 ng/mL	–	2021 [86]
	Prostate Cancer	Ag Nanogap Shell NPs	Antibody	SERS Spectroscopy	N/A	2 pg/mL	1.6–25 pM	–	2021 [87]
	Prostate Cancer	Au@Ag Core–Shell NPs	Aptamer	SERS Spectroscopy	Buffer	0.38 ag/mL	10^–2^–10^–15^ mg/mL	Serum (*n* = 5)	2021 [88]
	Prostate Cancer	QD-embedded Silica NPs	Antibody	LFA	Buffer	0.138 ng/mL	N/A	Plasma (*n* = 47)	2022 [89]
Neuron-Specific Enolase (NSE)	Lung Cancer	Graphene QDs and AuNPs	Antibody	Fluorescence Detection	N/A	0.09 pg/mL	0.1–1000 ng/mL	–	2020 [90]
	Lung Cancer	AgNPs/Ti_3_C_2_-MXene and GQDs	Antibody	Fluorescence Detection	N/A	0.05 pg/mL	0.0001–1500 ng/mL	–	2022 [91]
	Lung Cancer	Ag Nanodome	Antibody	Colorimetric Detection	Buffer	270 pM	N/A	–	2023 [92]
Hepatitis B Surface Antigen (HBsAg)	Hepatitis B and Hepatocellular Carcinoma	AuNRs	Antibody	LSPR	Buffer	0.01 IU/mL	0.01–1 IU/mL	Plasma (*n* = 6)	2010 [93]
	Hepatitis B and Hepatocellular Carcinoma	Au Nanoflower	Antibody	SERS Spectroscopy	Plasma	0.01 IU/mL	0.0125–60 IU/mL	–	2015 [94]
	Hepatitis B and Hepatocellular Carcinoma	AuNPs	Antibody	LSPR	N/A	100 fg/mL	10 pg/mL–10 ng/mL	–	2018 [95]
	Hepatitis B and Hepatocellular Carcinoma	Polystyrene Nanospheres	Antibody	Colorimetric Detection	Buffer	0.1 ng/mL	N/A	–	2021 [96]
				LSPR	Buffer	0.01 ng/mL	0.01–10 ng/mL		
Mouse Double Minute 2 Homolog (MDM2) Tau	Cancer	AuNPs	Aptamer	LSPR	N/A	20 nM	30–50 nM	–	2016 [97]
	Alzheimer's Disease	AuNPs	Antibody	LSPR	N/A	10 pg/mL	N/A	–	2008 [98]
	Alzheimer's Disease	AuNPs (+ Magnetic NPs)	Antibody	SERS Spectroscopy	Buffer	25 fM	25 fM–500 nM	–	2013 [99]
	Alzheimer's Disease	MWCNTs	Antibody	SPR Spectroscopy	Artificial CSF	125 pM	1–25 nM	–	2017 [100]
	Alzheimer's Disease	AuNRs	Antibody	LSPR	Plasma	100 fM	10^2^–10^8^ fM	–	2019 [101]
Tau-441	Alzheimer's Disease	AgNPs	Antibody	SERS Spectroscopy	Plasma	3.21 fM	10 fM–1 uM	–	2022 [102]
Tau-381	Alzheimer's Disease	Au Nanopopcorn	Aptamer	SERS Spectroscopy	N/A	2.2 fM	0.1 fM–1 nM	–	2023 [103]
Cardiac troponin I (cTnl)	Acute Myocardial Infarction	Au@AgNPs	Antibody	SERS Spectroscopy	N/A	9.80 pg/mL	0–2.0 mg/mL	Serum (*n* = 50)	2021 [104]
	Acute Myocardial Infarction	NaYF_4_:Yb,Tm@NaYF_4_ UCNPs	Antibody	ULISA	Plasma	0.13 ng/mL	N/A	–	2022 [105]
					Serum	0.25 ng/mL	6.7–77.8 ng/ml		
	Acute Myocardial Infarction	NaYF_4_:Yb^3+^, Tm^3+^@NaY–F_4_:Yb^3+^, Nd^3+^@NaYF_4_ UCNPs	Antibody	Upconversion Luminescence Detection	Buffer	0.24 fg/mL	1 fg/mL–100 pg/mL	–	2024 [106]

^a^The healthy donors’ biofluids, which are utilized to make model samples by spiking known concentrations of target analytes (e.g., recovery tests), are excluded here. To avoid confusion, we added only the biofluids obtained from actual patients (i.e., unknown samples) as “clinical samples” in this table

**Table 4:** The optical nanomaterial-based biosensors for the multiplexed detection of protein LB markers

Biomarker	Disease	Optical Nanomaterial	Biorecognition Element	Detection Method	Matrix	Limit of Detection	Linear Range	Clinical Sample [a]	Note
AFP	Lung Cancer	QDs	Antibody	Fluorescence Detection	N/A	250 fM	25 fM–250 nM	–	2010 [107]
CEA						250 fM	25 fM–250 nM		
CEA	Lung Cancer	QDs	Antibody	Fluorescence Detection	N/A	1.0 ng/mL	3–100 ng/mL	Serum (*n* = 25)	2011 [108]
NSE						1.0 ng/mL	3–100 ng/mL		
AFP	Lung Cancer	AuNPs	Antibody	LSPR	Serum	91 fM	10–10^6^ fM	–	2015 [109]
CEA						94 fM	10–10^6^ fM		
PSA						10 fM	10–10^6^ fM		
AFP	Liver Cancer	CdSe/ZnS QDs	Antibody	SPR Spectroscopy	Buffer	0.1 ng/mL	0.1–1000 ng/mL	–	2016 [110]
CEA	Colorectal Cancer					0.1 ng/mL	0.1–1000 ng/mL		
CYFRA 21–1	Lung Cancer					0.1 ng/mL	0.1–1000 ng/mL		
PSA	Cancer	SiNPs (w/ SiC@Ag Substrate)	Antibody	SERS Spectroscopy	N/A	1.79 fg/mL	10^–4^–10^–1^ ng/mL	Serum (*n* = 5)	2016 [70]
AFP						0.46 fg/mL	10^–4^–10^–1^ ng/mL		
CA 19–9						1.3 × 10^−3^ U/mL	10^–1^–10^3^ U/mL		
CEA	Lung Cancer	QDs	Antibody	Fluorescence Detection	N/A	38 pg/mL	3.9–125.0 ng/mL	–	2016 [111]
CYFRA 21–1						364 pg/mL	3.9–62.5 ng/mL		
NSE						370 pg/mL	3.9–62.5 ng/mL		
AFP	Cancer	AuNPs and UCNPs	Aptamer	SERS Spectroscopy	Buffer	0.059 aM	1–100 aM	–	2017 [112]
Mucin-1						4.1 aM	0.01–10 fM		
AFP	Cancer	Magnetic GQDs	Antibody	Fluorescence Detection	N/A	0.06 pg/mL	0.2–680 pg/mL	–	2017 [113]
CA-125						0.001 ng/mL	0.003–25 ng/mL		
AFP	Cancer	CdZnTeS QDs (+ Magnetic NPs)	Antibody	ECL Detection	Buffer	0.1 fg/mL	0.5–20 ng/mL	Serum (*n* = 3)	2018 [114]
CA-125						0.03 mU/mL	0.1–500 U/mL		
cTnl	Heart Failure	Au@AgNPs (+ Magnetic NPs)	Antibody	SERS Spectroscopy	N/A	0.6396 ng/mL	0–100 ng/mL	Serum (*n* = 50)	2020 [115]
Heart-type fatty acid binding protein						0.0044 ng/mL	0–1 ng/mL		
CEA	Cancer	QD-encoded Polymer Microsphere	Antibody	Fluorescence Detection	N/A	0.138 ng/mL	N/A	–	2022 [116]
CA-125						1.60 KU/L	N/A		
CA 19-9						0.92 KU/L	N/A		
CA 72-4						1.06 KU/L	N/A		
CA 125	Oral Cancer	AuNPs	Antibody	LSPR	Buffer	1.6 U/mL	5–320 U/mL	–	2022 [117]
CYFRA 21-1						0.84 ng/mL	0.496–48.4 ng/mL		
CEA	Cancer	Porous Au–Ag NPs	Antibody	SERS Spectroscopy	N/A	1.22 × 10^−8^ ng/mL	10^−7^–10^3^ ng/mL	–	2023 [118]
AFP						2.47 × 10^−5^ ng/mL	10^−4^–10^3^ ng/mL		

^a^The healthy donors’ biofluids, which are utilized to make model samples by spiking known concentrations of target analytes (e.g., recovery tests), are excluded here. To avoid confusion, we added only the biofluids obtained from actual patients (i.e., unknown samples) as “clinical samples” in this table

Xu et al. presented a novel immunoassay for the detection of AFP using a plasmon-induced silver photoreduction system [59]. The silver crystals were generated on the surface of AuNPs by only the visible light illumination without using reducing agents. Thanks to this enzyme-free amplification, the sensitivity of the sensor was largely enhanced in a simple manner. The LOD was 3.3 fg/mL, which is more than 3 orders of magnitude lower compared to the LOD of commercial ELISA (around 6 pg/mL).

Recently, more studies have adopted two or more nanomaterials to achieve synergetic effects. Wang et al. reported an aptasensor for the detection of CEA based on fluorescence resonance energy transfer (FRET) between UCNPs and GO [48]. When CEA was added, the structure of aptamer was changed, and UCNPs were separated from the GO, resulting in fluorescence recovery. The LOD was 7.9 pg/mL in aqueous solution and 10.7 pg/mL in serum, and it is almost 2 orders of magnitudes lower than the LOD of commercial ELISA mentioned above (around 0.2 ng/mL). Li et al. developed a hybrid SERS immunosubstrate consisting of Au nanoflowers and red phosphorus (RP) nanoplates [73]. The anisotropic growth of 3D NPs having sharp edges on the 2D RP substrate, which is advantageous in electron conductivity and visible-light-responded bandgaps, provides a sensitive and robust platform. The LOD of the sensor was 7.41 × 10^–5^ U/mL and it is much lower than the cut-off value (37 U/mL) and the LOD of the commercial ELISA kits (around 0.3 U/mL). More importantly, the presented immunosubstrates were recyclable through the photocatalytic degradation of antigens and antibodies.

Medetalibeyoglu et al. utilized three different types of nanomaterials to develop sensitive and selective SERS-based sandwich immunoassays [62]. In this design, 2-dimensional transition metal dichalcogenides (TMDCs) and AuNPs are hybridized to prepare SERS probes. In the meantime, Ti_3_C_2_T_x_ MXenes and Fe_3_O_4_ NPs@Au NPs are incorporated to fabricate SERS substrates. It is one example of the rational design of nano material-based immunoassay because metal NPs cover the limited efficiency and low functionality of TMDCs. At the same time, 2-dimensional materials like TMDCs can provide chemical enhancement to AuNP-based systems. MXenes, another 2-dimensional nanomaterial, also provide similar advantages, and the incorporation of Fe_3_O_4_ NPs@Au NPs makes the resulting sheets into magnetic substrates for the enhancement of sensitivity and specificity via magnetic separation. As a result, the system showed 0.033 pg/mL of LOD and a wide dynamic range covering 6 orders of magnitudes.

Another important direction of protein LB biomarkers is multiplexed detection. The simultaneous measuring of two or more biomarkers from identical samples can clarify the complex relationship between biomarkers and disease, so eventually, it may provide the opportunity for early detection. The simultaneous detection of multiple biomarkers is demonstrated by two different combinations: (a) representative biomarkers but not limited to specific cancer; (b) clinically related biomarkers of s single cancer subtypes.

Lee et al. proposed an example of the former concept. Their nanoplasmonic biosensor based on AuNPs targeted AFP, CEA, and PSA, and those protein biomarkers are renowned indicators of liver, lung, and prostate cancers. The LOD of the sensor was 91 fM, 94 fM, and 10 fM for AFP, CEA, and PSA, respectively, from the serum samples. These are much lower levels than both cut-off values (picomolar level) and even the background of healthy individuals. In addition, a wide dynamic range, from the femtomolar level to the nanomolar level, traces the changes over the biological range; therefore, this kind of approach may contribute to the early screening of the potential disease. On the other hand, Wu et al. designed an example of the latter concept, particularly focusing on lung cancer. Their sandwich immunoassay using multicolor QDs and magnetic microbeads targeted three protein biomarkers (CYFRA 21-1, CEA, and NSE) for lung cancer. Thanks to the different colors of three types of QDs, which are designated three different biomarkers, the concentration of each or the ratio between them was evaluated. The LOD of the sensor was 38 pg/mL, 364 pg/mL, and 370 pg/mL for CYFRA 21-1, CEA, and NSE, respectively. This kind of approach may contribute to the accurate detection of lung cancer regardless of the concentration of a specific biomarker. In addition, the diagnosis based on multiple biomarkers provides valuable information for future treatment decision-making.

### Peptide

Peptides are short chains of amino acids that are linked via peptide bonds [119]. Like proteins, peptides also are amino acid-based building blocks in living organisms. The difference between them is size and structure, thus rendering distinct biological functions [120]. When the liquid biopsy was first introduced, it mainly focused on oncology because its concept was a counterpart of tissue biopsy. Later, its range has been expanded to other diseases that can find biomarkers from the biofluids. The most famous peptide biomarkers are amyloid-beta (1–40) and amyloid-beta (1–42), which have long been considered as biomarkers of Alzheimer’s disease (AD) [121]. Historically, much effort has been made to detect these peptides, as well as tau protein, from cerebrospinal fluid (CSF) and even plasma in advance of the diagnosis by the medical imaging system. Detecting AD biomarkers is helpful in early diagnosis, and early diagnosis is beneficial in disease management and treatment. In the past, this idea had never been described as a “liquid biopsy.” For now, more and more literature set the expanded range of liquid biopsy, including biomarkers of other diseases [122, 123]. Although the CSF, a special kind of biofluid that cannot be accessible without an invasive procedure, is not perfectly fit for the philosophy of liquid biopsy, amyloid-beta in plasma or other fluid is more matched to the concept of liquid biopsy. The difficulty in detecting amyloid-beta is a relatively high background level that is not differentiated between the patient group and the control group.

Another peptide biomarker can be found in the field of cardiovascular diseases (CVD). Like AD biomarkers, CVD biomarkers had not been considered the LB biomarkers in the past; but more recent articles have started to discuss CVD detection as a part of liquid biopsy [124]. The most widely used CVD markers are brain natriuretic peptide (BNP) and N-terminal proBNP (NT-proBNP). They are significant indicators in heart failure and cardiac dysfunction. These peptides are secreted from the walls of the heart chamber directly into the bloodstream. The clinical cut-off of BNP and NT-proBNP is 100 pg/mL and 300 pg/mL, respectively [30], but the background level usually increases in the older age groups [125]. Various commercial test kits with analyzers for detecting peptide biomarkers have recently been on the market. For example, Roche Elecys® (Roche diagnostics) is one of the widely used methods in clinics to test BNP and NT-proBNP. This electrochemiluminescence immunoassay (ECLIA)-based system displays a high degree of diagnostic accuracy. With an 18-min testing time, the system is capable of detecting NT-proBNP as low as 5 pg/mL. Roche Elecys® systems are also developed for amyloid beta peptide detection. It shows 90% of concordance with amyloid PET imaging [126].

The representative cases of peptide LB marker detection using optical NPs are described in Table 5. The NPs discussed in these studies are metallic NPs, silica-coated metallic NPs, and MWCNTs. Among peptide LB biomarkers, the detection of BNP and pro-BNP are relatively similar to that of protein LB biomarkers. Because there already is a confirmed reference level in specific biofluids, setting a guideline for detecting them is relatively clear. The performance of the developed methods needs to be sensitive and accurate around the cut-off levels. Most studies report a better LOD than the general cut-off level of BNP (100 pg/mL) and proBNP (300 pg/mL) and even find a way to reach a sub-picogram level for early detection.

**Table 5:** The optical nanomaterial-based biosensors for the detection of peptide LB biomarkers

Biomarker	Disease	Optical nanomaterial	Biorecognition element	Detection method	Matrix	Limit of detection	Linear range	Clinical sample [a]	Note
Beta-Amyloid (1–42)	Alzheimer's Disease	AuNPs	N/A	LSPR	CSF	1.5 pM	N/A	–	2015 [127]
	Alzheimer's Disease	QDs (+ Magnetic Beads)	Antibody	Fluorescence Detection	Buffer	0.2 nM	0.5–8.0 nM	–	2016 [128]
	Alzheimer's Disease	AuNPs	Antibody	Colorimetric Detection	Buffer	2.3 nM	7.5–350 nM	–	2017 [129]
	Alzheimer's Disease	QDs	Antibody	Fluorescence Detection	Diluted CSF	1.7 pM (7.6 pg/mL)	5–100 pM (0.023–0.45 ng/mL)	–	2018 [130]
	Alzheimer's Disease	Pt@Au Triangular Nanorings	N/A	SERS Spectroscopy	Buffer	0.045 pM	0.1–1000 pM	CSF (*n* = 5)	2021 [131]
	Alzheimer's Disease	Au@AuNPs	N/A	SERS Spectroscopy	Salt-Containing Solution	650 pg/mL	0.04–8 ng/mL	–	2023 [132]
					CSF	124 pg/mL	347–629 pg/mL		
Beta-Amyloid (1–40)	Alzheimer's Disease	AuNPs	Antibody	LSPR	Buffer	34.9 fM	10^1^–10^8^ fM	–	2018 [133]
Beta-Amyloid (1–42)			Antibody	LSPR	Buffer	26.0 fM	10^1^–10^8^ fM		
Beta-Amyloid (1–40)	Alzheimer's Disease	Si@Ag NPs (+ Magnetic Beads)	Antibody	SERS Spectroscopy	Buffer	0.25 pg/mL	N/A	–	2019 [134]
Beta-Amyloid (1–42)			Antibody	SERS Spectroscopy	Buffer	0.33 pg/mL	N/A		
Beta-Amyloid Fibrils	Alzheimer's Disease	QDs	Benzotriazole (BTA)	Fluorescence Detection	Artificial CSF	45 pM	1 uM–20 uM	–	2016 [135]
		Pt@Au Triangular Nanorings	N/A	SERS Spectroscopy	Buffer	4 fM	0.1–1000 pM	CSF (*n* = 5)	2021 [131]
Beta-Amyloid	Alzheimer's Disease	AuNPs	N/A	Fluorescence Detection	CSF	100 fg/mL	0.61–1 ng/mL	–	2017 [136]
Beta-Amyloid Oligomer	Alzheimer's Disease	AuNPs	Antibody	Fluorescence Detection	Media	22.3 pM	0.1–1.0 nM	–	2020 [137]
	Alzheimer's Disease	UCNPs (NaYF_4_:Yb^3+^,Er^3+^)	Zinc Zeolitic Imidazole Framework	Fluorescence Detection	Buffer	28.4 pM	100 pM–10 uM	–	2021 [138]
	Alzheimer's Disease	AgNPs	N/A	SERS Spectroscopy	Salt-Containing Solution	15 pM	10^−8^ − 10^−4^ M	–	2023 [139]
Brain Natriuretic Peptide (BNP)	Heart Failure	AuNPs	Antibody	SPR Spectroscopy	Buffer	25 pg/mL	10^2^–10^3^ pg/mL	–	2006 [140]
N-terminal proBNP (NT-proBNP)	Heart Failure	AuNRs and MWCNTs	Antibody	ECL Detection	Plasma	3.86 fg/mL	0.01 − 100 pg/mL	–	2015 [141]
	Heart Failure	CoFe_2_O_4_@Au NPs and MOF-3@Au Tetrapods	Antibody	SERS Spectroscopy	N/A	0.75 fg/mL	0.001 − 1000 pg/mL	–	2016 [141]
	Heart Failure	UCNPs (NaYF_4_:Yb^3+^,Er^3+^)	Antibody	LFA	Buffer	116 ng/L	50–35,000 ng/L	Blood/Serum (*n* = 91)	2017 [142]
	Heart Failure	MoS_2_@Cu_2_S-Au and MZnAgInS/ZnS@MOF Nanocrustals	Antibody	ECL Detection	Buffer	0.41 fg/mL	1 fg/mL–100 ng/mL	–	2020 [143]
	Heart Failure	Covalent Organic Framework@AuNPs (+ Magnetic NPs)	Antibody	Dynamic light scattering (DLS)	Diluted Blood (1/20)	14 fg/mL	0.32–1000 pg/mL	–	2022 [144]

^a^The healthy donors’ biofluids, which are utilized to make model samples by spiking known concentrations of target analytes (e.g., recovery tests), are excluded here. To avoid confusion, we added only the biofluids obtained from actual patients (i.e., unknown samples) as “clinical samples” in this table

On the other hand, another type of peptide LB biomarker, like beta-amyloid, is far more complicated. The absence of enough clinical evidence and somewhat contracted reports among the studies are problematic when setting a guideline for the detection of beta-amyloid. The concentration range of these peptides is broadly distributed with individual differences, and even background level keeps increasing along with normal aging. According to the previous studies, the lower limit of plasma concentration of Aβ_(1–40)_ and Aβ_(1–42)_ is 10^–11^ and 10^–12^ g/mL, respectively [34]. In the case of beta-amyloid, multiplexed detection of Aβ_(1–40)_ and Aβ_(1–42)_ is an essential requirement because the ratio between them is more prominent than each concentration [145]. Kim et al. [133] suggested a shape-code plasmonic biosensor for the detection of three kinds of AD biomarkers, Aβ_(1–40)_, Aβ_(1–42)_, and tau proteins. Each biomarker was coded using 50 nm AuNPs, AuNRs (aspect ratio = 1.6), and AuNRs (aspect ratio = 3.6), respectively. The LOD of the sensor for each biomarker was 34.9 fM, 26.0 fM, and 23.6 fM, respectively. These are much lower levels compared to the background concentration of these biomarkers. Therefore, the presented one-step multiple detection offers an opportunity for sensitive and accurate detection of AD biomarkers. In the meantime, Yang et al. presented SERS-based multiplexed detection of Aβ_(1–40)_ and Aβ_(1–42)_ using silver nanogap shells on Si NPs and magnetic beads [134]. In the format of sandwich immunoassay, LOD was 0.25 pg/mL and 0.33 pg/mL for Aβ_(1–40)_ and Aβ_(1–42)_, respectively. This performance based on the intense and stable SERS signals also indicates the detection of a very low amount of biomarkers from the complex matrix like serum. Further, Wang et al. fabricated Pt@Au plasmonic chiral triangular nanorings to detect both Aβ_(1–42)_ monomers and fibrils [131]. Based on the intense chiral response of triangular nanorings modified with L- and D-glutathione, the proposed methods took advantage of the SERS-chiral anisotropy effect. The LOD of the system was 0.045 × 10^–12^ M and 4 × 10^–15^ M for monomer and fibrils, respectively. This study provides the opportunity to investigate the process of amyloid peptide misfolding and aggregation.

Aβ oligomers (AβO) are one of the important themes in AD research. Fang et al. reported a detection method for AβO based on fluorescence ratio using ZIF-8-doped UCNPs-SiO_2_@metal–organic framework/black hole quencher [138]. The authors utilized optical tweezer microscopic imaging. It is an interesting approach because optical trapping prevents interference with fluid viscosity. The microsphere embedding nanomaterials are advantageous in both marker enrichment and laser focusing. The LOD of the sensors was 28.4 pM, and quantitative detection was demonstrated between 100 pM and 10 μM. Yin et al. designed a 3-dimensional fluorophore-labeled DNA walker nanoprobe immobilized on the AuNPs [137]. These nanoprobes can detect AβO and provide real-time imaging in living cells and in vivo. When the AβO was present in the samples, the fluorophores were cleaved and released, thus enabling a signal amplification effect without enzyme. Under in vitro demonstration, LOD was 22.3 pM, and the dynamic range was confirmed in the concentration range of 0.1 to 1.0 nM.

### Circulating tumor DNAs

Circulating tumor DNAs are tumor-derived fractions of cell-free DNAs (cfDNAs) [146]. Although the amount of cfDNAs fluctuated in healthy individuals, an elevated level of cfDNA in cancer patients was found in the early studies. Leon et al. reported that the plasma concentration of cfDNA in healthy control was in the range between 0 and 100 ng/mL (mean = 13 ± 3 ng/mL) [38]. On the other hand, the concentration of cfDNA in cancer patients was highly varied from 0 to 5000 ng/mL (mean = 180 ± 38 ng/mL). Interestingly, there was a huge disparity between the upper 50 percent and lower 50 percent, and this result indicates that the cfDNA level is usually high in cancer patients.

Currently, there are two approaches to detecting mutations in ctDNA [147]. The first one is a targeted detention using complementary oligonucleotides. Because this approach mainly focused on the known mutations in specific genes, the patients who do not have these mutations cannot be distinguished. Conversely, the second one is untargeted detection based on next-generation sequencing (NGS). This approach sequences millions of DNA fragments at the same time via the “sequencing by synthesis” method, so a large amount of information can be obtained, including unknown mutations. Therefore, it is a time-consuming procedure conducted by highly trained experts, and it also generates an extensive volume of data requiring elevated costs [148]. For these reasons, many efforts have been made to develop a sensitive detection system comparable to an NGS-based assay. For example, Nesvet et al. developed magnetic NP-based giant magnetoresistive sensors that detect 0.01% mutant allelic fraction in ctDNA. It achieved both high analytical sensitivity and rapid testing time [149].

Especially, ctDNAs recently gained more attention than CTCs due to the advances in sequencing technology and relatively simple preprocessing procedures. For example, Grail Inc. developed NGS-based ctDNA detection tests (“Galleri”) for multi-cancer early screening [150]. They have constructed the mutation library from large-scale discovery to distinguish the usual mutations and tumor-related mutations.

However, there are difficulties in ctDNA detection. First, the actual fraction of ctDNA is extremely low, like other LB biomarkers. There is a report that around 1% to 2% of overall cfDNA are accounted for ctDNA in cancer patients [151]. Second, its status is highly varied due to the short half-life and thus dependent on the sampling moment [152]. It implies the possibility of real-time monitoring of the tumor; but. It also is a technical huddle in developing sensing methods. Third, a large-volume sample is usually required to reach a satisfactory sensitivity [153]. These issues are getting even worse in the case of circulating free RNAs or circulating tumor RNAs, more rare and more unstable targets. In order to overcome this limitation, the detection of nucleic acid LB biomarkers requires both rapid and ultrasensitive sensing mechanisms.

In the biosensing field, ctDNA detection methods are basically based on the historical achievement in aptasensors [154, 155]. Therefore, the specific direction of the research has been tuned to find a disease-related sequence in cfDNAs. Key mutations like rat viral sarcoma (*RAS*), *EGFR*, *PIK356*, *BRAF*, and *TP53* were targeted to estimate the actual fraction of ctDNA from total cfDNA [156–158]. The representative studies using optical nanomaterials are described in Table 6. The lowest LOD is down to the attomolar range, and the widest linear range was 5 orders of magnitudes. Although these results cover the concentration range of cfDNA in plasma (Table 1), it is still hard to estimate the actual concentration of the mutated cfDNAs.

**Table 6:** The optical nanomaterial-based biosensors for the detection of circulating free DNAs or circulating tumor DNAs

Biomarker	Disease	Optical nanomaterial	Biorecognition element	Detection method	Matrix	Limit of detection	Linear range	Clinical sample [a]	Note
ctDNA (*PIK3CA* Mutation)	Cancer	AuNPs	PNA	LSPR	Serum	50 fM	50–3200 fM	–	2015 [159]
ctDNA (*KRAS/PIK3CA* Mutation)	Cancer	CuNPs (+ SWNTs)	Triple-Helix Molecular Switch (THMS)	SERS Spectroscopy	Buffer	1.5 fM	10 fM–1 nM	Serum (*n* = 6)	2016 [160]
ctDNA (Methylation)	Cancer	AuNPs and AgNPs (+ Graphene)	Antibody	SERS Spectroscopy	N/A	0.2 pg/uL	0.05 ng/uL–5 ng/uL	–	2017 [161]
ctDNA (*EGFR* Mutation)	Cancer	AuNPs	Complementary DNA	Colorimetric Detection	N/A	7.7 fM	870 aM–87 pM	–	2018 [162]
ctDNA	Cancer	Silica-Coated Au Nanorods	Complementary DNA	SERS Spectroscopy	Buffer	57.74 nM	100 nM–1000 nM	–	2019 [163]
ctDNA (*KRAS* Mutation)	Cancer	AuNCs and UCNPs (NaYF_4_:Yb^3+^, Er^3+^)	Complementary DNA	Fluorescence Detection	Serum	6.30 pM	5 pM–1000 pM	–	2020 [164]
cfDNA (*RAS* Mutation)	Colorectal Cancer	AuNPs	PNA	SPR Imaging	N/A	N/A	N/A	Blood (*n* = 12)	2020 [165]
ctDNA	Cancer	QDs	THMS	Fluorescence Detection	Plasma	5.4 pM	10 pM–100 pM	–	2021 [166]
cfDNA (*KRAS* Mutation)	Colorectal Cancer	AuNPs	PNA	SPR Imaging	Plasma	2.5 aM	0.5 − 20.0 pg/ μL	Plasma (*n* = 1)	2021 [167]
ctDNA (*EGFR* Mutation)	Lung Cancer	AuNPs and Graphitic-Carbon Nitride QDs (g-CNQDs)	Complementary DNA	ECL-RET	Buffer	0.00055 fM	0.001 fM–1 pM	–	2021 [168]
					Plasma	0.0023 fM	0.01 fM–1 pM	–	
ctDNA (CYFRA21-1 Mutation)	Lung Cancer	QDs (+ Magnetic NPs)	Complementary DNA	Fluorescence Detection	N/A	53 aM	1 fM–1 nM	–	2022 [169]
cfDNA (*TP53* and *PIK356* Mutation)	Lung Cancer	Au–Ag Nanoshuttle	Complementary DNA	SERS Spectroscopy	Serum	2.26 aM *(TP53)*	10 aM–100 pM	Serum (*n* = 120)	2022 [170]
						2.34 aM *(PIK356)*	10 aM–100 pM		
ctDNA (BRAF and *KRAS* Mutation)	Lung Cancer	Pd-Au Core–Shell Nanorods (+ Magnetic Beads)	Complementary DNA	SERS Spectroscopy	Buffer	3.116 aM *(BRAF)*	10 aM–100 pM	–	2022 [171]
					Mouse Serum	4.257 aM *(BRAF)*	10 aM–100 pM		
					Buffer	3.921 aM *(KRAS)*	10 aM–100 pM		
					Mouse Serum	6.183 aM *(KRAS)*	10 aM–100 pM		
ctDNA (*EGFR* Mutation)	Lung Cancer	MnO_2_ nanosheets and Fluorescent Polydopamine NPs	Complementary DNA	SERS Spectroscopy	Buffer	380 pM	25–125 nM	–	2023 [170]
ctDNA (*EGFR* Mutation)	Lung Cancer	AuNPs and CdS QDs	Complementary DNA	ECL-RET	Buffer	8.1 aM	10 aM–100 fM	–	2023 [172]
					Plasma	91 aM	100 aM–1 pM	–	

^a^The healthy donors’ biofluids, which are utilized to make model samples by spiking known concentrations of target analytes (e.g., recovery tests), are excluded here. To avoid confusion, we added only the biofluids obtained from actual patients (i.e., unknown samples) as “clinical samples” in this table

There are several criteria for the evaluation of ctDNA detection techniques. First of all, ultrasensitive detection and accurate quantification are essential, considering the minuscule amount of ctDNAs in the blood. Unlike traditional LB biomarkers, the sensitivity of the system is required to be down to a single-nucleotide level, distinguishing point mutation precisely and accurately. Eventually, simultaneous detection of multiple genetic mutations is a significant criterion for maximizing clinical feasibility.

Several studies reported multiplexed ctDNA detection technologies to conform to two kinds of point mutations simultaneously. Nguyen et al. present a strategy for the dual detection of ctDNAs via targeting two bio-signatures, E542K and E545K, tumor-specific genetic and epigenetic markers of ctDNA of *PIK3CA* gene [159]. The probe was designed using AuNPs functionalized with peptide nucleic acids (PNA). The capture and the enrichment of ctDNA induced the change of reflective index and can be detected as the peak change of LSPR. Moreover, the authors utilized the coupling plasmon mode to detect both epigenetics changes and enhanced the signal of specific genetic mutations. SERS-based ctDNA detection was also demonstrated by the advances in SERS immunoprobes and/or SERS immune-substrates. Lin et al. developed a SERS-active substrate for the detection of tumor-related DNAs. With a dual signal amplification method, using metal carbonyls (metal-COs) onto SiO_2_@Au as interference-free SERS labels, the LOD of the system was 57.74 M, and the linear range is between 100 and 1000 nM. Bellassai et al. investigated ctDNA detection using SPR imaging systems [167]. The sensor interface, poly-L-lysine (PLL)-based dual functional layer, was designed to achieve two purposes: anti-fouling surface and immobilization of PNA probes. The sensor detects wild-type and Kirsten rat viral sarcoma (*KRAS*) p.G12D- and p.G13D-mutated genomic DNAs in plasma. The LOD of the sensor was 5 pg/μL level and it is equivalent to approximately 2.5 aM. It does not require preprocessing for DNA isolation and PCR amplification.

Cao et al. developed pump-free SERS microfluidic chips to detect both *BRAF* V600E-mutated and *KRAS* G12V-mutated ctDNAs [171]. The identification of *BRAF* V600E mutation, which is discovered in 3% of non-small cell lung cancer, is important in the decision of therapy. Likewise, the identification of *KRAS* mutation is related to poor survival rate. Therefore, simultaneous quantification of both mutations from ctDNA provides detailed information about the characteristics of the primary tumor. The authors especially combined SERS nanoprobe (Pd-Au nanorod@magnetic bead), catalytic hairpin assembly, and microfluidics. The high sensitivity of this study is derived from a dual-signal amplification strategy, CHA-based amplification, and magnetic beads-based aggregation. The LOD was 3.116 aM and 3.921 aM for *BRAF* V600E and *KRAS* G12V, respectively. Further, the authors confirm that attomolar level sensitivity and accurate quantification are present in mouse serum. Later, the authors proposed another micro fluid-based platform for evaluating expression levels of *TP53* or *PIK3CA*-Q546K in ctDNAs [170]. *TP53* is usually considered to be related to a worse prognosis and resistance to chemotherapy, and *PIK3CA*-Q546K plays an important role in the pathogenesis of NSCLC. The design of this study is basically similar to the previous one, but Au–Ag nano shuttles were utilized as SERS nanoprobes, and the Au–Ag nano bowl array was prepared as SERS substrates. The LOD was 2.26 aM and 2.34 aM for *TP53* and *PIK3CA*-Q546K, respectively. Finally, the clinical feasibility was verified by comparing with qRT-PCR tests, using patients’ samples and healthy donors’ samples.

### microRNAs

In the same context, extracellular RNAs in the biofluid can also be a potential biomarker. Almost all kinds of them are released through the death of the cells or the active release mechanism of the cells [173]. However, they are extremely unstable, and their half-life is estimated to be just a few seconds, so most RNA-related liquid biopsy studies tend to focus on the complexed form with the proteins or encapsulated form in the exosomes [174]. Among them, miRNAs are the most notable biomarkers in the RNA family. These nonprotein-cording RNAs, having a length of 19 to 25 nucleotides, are relatively stable compared to other nucleic acids [175]. In addition, the expression of these post-transcriptional regulators for gene expression is presumed to be dysregulated in various cancers. Because miRNA expression levels in blood have correlated with miRNA levels in tumor tissue, monitoring its level in blood can be a feasible approach to liquid biopsy [176]. Unfortunately, the evaluation of miRNA expression level has similar issues to other LB biomarkers. Current methods, including Northern blotting and RT-PCR, require complex and time-consuming procedures. There also is a risk of contamination. More importantly, high sensitivity is required to monitor the changes derived from diseases.

The representative studies using optical nanomaterials are described in Table 7. The lowest LOD is down to the femtomolar range, and the widest linear range was 10 orders of magnitude. There are several criteria for the evaluation of miRNA detection techniques. First, ultrasensitive detection and wide dynamic range are key criteria. Zhu et al. developed ECL biosensors for the detection of miR-182 [177]. The miRNAs were successfully separated and detected via enzymatical enhancement with the combination of the AuNP-decorated magnetic particles and QD-embedded mesoporous silica nanoparticles. The LOD of the sensor was 33 fM, and the linear range was in the range between 100 fM and 100 pM.

**Table 7:** The optical nanomaterial-based biosensors for the detection of microRNA

Biomarker	Disease	Optical nanomaterial	Biorecognition element	Detection method	Matrix	Limit of detection	Linear range	Clinical sample [a]	Note
miRNA (miR-141)	Cancer	Au Nanocubes	DNA Probe	Fluorescence Detection	Buffer	2 aM	1 aM—1000 pM	–	2012 [178]
miRNA (miR-21, miR-155)	Breast Cancer Ovarian Cancer	AuNPs	DNA Probe	SERS Spectroscopy	N/A	1 nM	1 nM–10 nM	–	2017 [179]
miRNA (miR-155)	Breast Cancer	CdTe QDs	DNA Probe	Fluorescence Detection	Buffer	0.42 pM	10 pM–100 pM	–	2018 [180]
miRNA (miR-34a)	Gastric Cancer	Ag Nanocrystals in Au Nanobowls	DNA Probe	SERS Spectroscopy	Buffer	1 fM	1 fM–1 nM	–	2018 [181]
miRNA (miR-10b miR-21 miR-373)	Breast Cancer	Head-Flocked Au Nanopillar	DNA Probe	SERS Spectroscopy	Serum	3.53 fM	10^−1^ fM–10^9^ fM	–	2019 [182]
						2.17 fM	10^−1^ fM–10^9^ fM		
						2.16 fM	10^−1^ fM–10^9^ fM		
miRNA (miR-182)	Lung Cancer	Fe_3_O_4_@Au and mSiO_2_@CdTe NSs	DNA Probe	ECL Detection	Buffer	33 fM	0.1 pM–100 pM	Serum (*n* = 3)	2019 [177]
miRNA (miR-21 miR-155 miR-16)	Breast Cancer	AuNPs PtNPs AgNPs (+ Magnetic Beads)	DNA Probe	Single-Particle Inductively Coupled Plasma-Mass Spectrometry (ICP-MS)	N/A	1.1 pM	10 − 300 pM	Serum (*n* = 14)	2022 [183]
						1.1 pM	10 − 300 pM		
						1.2 pM	10 − 200 pM		
miRNA (miR-224)	Liver Cancer	Au nanoarrays	DNA Probe	SERS Spectroscopy and Fluorescence Detection	Buffer	0.34 fM 0.39 fM	1 fM–1 nM	Serum (*n* = 16)	2023 [184]
miRNA (miR-375)	Prostate Cancer	AuNPs	DNA Probe	Plasmon-Enhanced Digital Imaging	Buffer	1.29 fM	1 fM–10 pM	–	2023 [185]

^a^The healthy donors’ biofluids, which are utilized to make model samples by spiking known concentrations of target analytes (e.g., recovery tests), are excluded here. To avoid confusion, we added only the biofluids obtained from actual patients (i.e., unknown samples) as “clinical samples” in this table

One of the approaches to overcome the limitations of the singular sensing mechanism is dual-mode sensing. Huang et al. developed SERS/Fluorescence biosensors consisting of well-arranged Au nanoarray substrates [184]. With the CHA-based amplification using fluorophore-labels hairpin DNAs, a stable, reliable, and reproducible signal was obtained from the miRNA assay. Based on the system integrating SERS and MEF effects, the authors detect HCC-related miR-224. The LOD of the system was 0.34 fM and 0.39 fM for SERS mode and fluorescence mode, respectively. The linear range was ranged from 1 fM to 10 nM via a triple enhancement system. In the validation using clinical samples obtained from the HCC patients, the level of miR-224 was largely reduced after hepatectomy.

Second, multiplexed detection is also essential in miRNA sensing technologies. Jiang et al. established the nanoparticle-based sandwich assay for the simultaneous detection of multiple miRNAs [183]. The authors separately encoded miR-21, miR-155, and miR-16 with AuNPs, PtNPs, and AgNPs, respectively, and collected via magnetic separation. The first two miRNAs are breast cancer-associated oncogenic miRNAs, whereas the last one is endogenous control. The results were analyzed by single-particle ICPMS, which can distinguish the signal differences derived from individual nanoparticles. The LOD of the sensor is 1.1 pM, 1.1 pM, and 1.2 pM for miR-21, miR-155, and miR-16, respectively, without requiring amplification steps.

### Exosomes

Extracellular vehicles (EVs) are lipid-bounded particles that are involved in intercellular communications [186]. Because they mirror the mother cells and thus carry proteins and nucleic acids originating from the mother cells, there has been a hypothesis of their physiological and pathological roles. EVs are usually classified by their mechanism in biogenesis, concept, and characteristics (e.g., size). Among them, exosomes are endosome-originated nanosized vesicles that are secreted from the cells and circulate until reaching recipient cells [187]. They are considered signaling molecules involved in cell-to-cell communications. Unlike the other three members, exosomes are abundant in concentration, from 10^7^ to 10^9^ particles per milliliter of plasma [188]. Because the cellular origin defines their composition, the ultimate objective of CTC or ctDNA research is also achievable by the strategy of detecting exosomes [189]. In addition, exosomes have advantages over CTCs or ctDNAs. First of all, they are plentiful in amount and the only LB biomarker free from rarity issues. Second, they are covered by a double-layered membrane and thus are considered a sort of cargo containing a package of nucleic acids (DNA, mRNA, and miRNA) and proteins. For these reasons, there have been efforts to isolate exosomes with other rare markers (CTCs and ctDNAs) simultaneously from identical samples to gather more information and to enhance the feasibility of the test [10]. Unfortunately, little is known about their characteristics and mechanisms. In the aspect of engineering, exosomes are hard-to-collect targets due to their broad size range and various surface markers. Furthermore, there is a purity issue because almost all cells generate exosomes. Therefore, the enrichment methods to separate tumor-derived fractions from the normal cell-derived vesicles are essentially required. The concentration of exosomal protein and exosome itself is relatively high in patients’ plasma compared to the blood obtained from healthy plasma [190].

Exosomes are the strangest targets in liquid biopsy research. Unlike other LB biomarkers, extracellular vesicles have never been an interest of the biosensing field before the liquid biopsy era. Therefore, there were no precedent schemes like aptasensors and cytosensors. Until now, various enrichment and isolation methods have been introduced, including ultracentrifugation, polymer-based precipitation, immunoaffinity-based separation, and acoustic-based purification [10, 191–193]. There also have been demonstrations using various optical detection methods. The representative studies are described in Table 8. Currently, several studies have reported approximately 10^3^ particles per mL, even down to around 10^2^ exosomes per mL. However, the results of these previously reported studies are hard to analyze systemically due to the ambiguousness of exosomes. All procedures, from sample preparation to final identification, are not established yet. For example, most exosome separation methods cannot guarantee that the impurities are negligible, so there is a possibility that the particles having similar characteristics to exosomes can be counted. For these reasons, it is difficult to set a minimally required sensing performance for exosome detection.

**Table 8:** The optical nanomaterial-based biosensors for the detection of circulating exosomes

Biomarker	Disease	Optical nanomaterial	Biorecognition element	Detection method	Matrix	Limit of detection	Linear range	Clinical sample [a]	Note
Exosome	Ovarian Cancer	Au NPs Au Nanostar	Antibody	SPR Spectroscopy	N/A	3000 exosomes	N/A	Ascites (*n* = 20)	2014 [194]
Exosome	Breast Cancer	Au@Ag Nanorods	Antibody	SERS Spectroscopy	Buffer	1200 exosomes/mL	– 10^5^ exosomes	–	2016 [195]
Exosome	Lung Cancer	Au Nanoisland	Antibody	LSPR	Serum	0.194 μg/mL	0.194–100 µg/mL	–	2017 [196]
Exosome	Breast Cancer	Single-Walled Carbon Nanotubes	fluorophore (FAM) labeled aptamer	Colorimetric Detection	Buffer	5.2 × 10^5^ particles/μL	1.84 × 10^6^–2.21 × 10^7^ particles/μL	Serum (*n* = 2)	2017 [197]
Exosome	Breast Cancer	MB@SiO_2_@AuNPs	Aptamer	SERS Spectroscopy	Buffer	32 exosomes/μL	10^2^–10^5^ exosomes	Blood (*n* = 1)	2018 [198]
	Colorectal Cancer					74 exosomes/μL	10^2^–10^5^ exosomes		
	Prostate Cancer					203 exosomes/μL	10^2^–10^5^ exosomes		
Exosome	Liver Cancer	UCNPs and AuNPs	Aptamer	Luminescence Imaging	Buffer	1.1 × 10^3^ particles/μL	10^4^–10^8^ particles/μL	–	2018 [199]
Exosome	Breast Cancer	AuNPs	Aptamer	SPR Spectroscopy	Serum	5 × 10^3^ exosomes/mL	N/A	–	2019 [200]
Exosome	Pancreatic Cancer, Colorectal Cancer, Bladder Cancer	AuNPs (+ Magnetic NPs)	Antibody	SERS Spectroscopy	Buffer	2.3 × 10^3^ particles/μL	N/A	–	2020 [201]
Exosome	Prostate Cancer	Magnetic NPs	Antibody	SERS Spectroscopy	Buffer	1.6 × 10^–1^ particles/μL	1.6 × 10^2^–1.6 × 10^9^ particles/mL	Serum (*n* = 8)	2020 [202]
Exosome	Gastric Cancer	UCNPs and AuNPs	Aptamer	ICP-MS	Buffer	0.074 μg/mL (4.7 × 10^3^ particles/mL)	0.5–6.0 μg/mL	Serum (*n* = 6)	2021 [203]
Exosome	Pancreatic Cancer	AuNPs and Polymer Dots	Antibody	ECL Detection	Buffer	400 particles/mL	10^3^–10^6^ particles/mL	Serum (*n* = 3)	2021 [204]
Exosome	Breast Cancer	AuNPs	Aptamer	SPR Spectroscopy	Buffer	1.0 × 10^4^ particles/mL	10^4^–10^7^ particles/mL	Serum (*n* = 8)	2021 [205]
Exosome	Not Specified	QD-Embedded Silica-Encapsulated NPs	Antibody	LFA	Buffer	117.94 exosome/µL	100–1000 exosome/µL	–	2022 [206]
Exosome	Liver Cancer	AuNPs and Zn-MOFs	CD63-Binding Peptide	ECL Detection	Buffer	9.08 × 10^3^ particles/μL	1.00 × 10^4^ − 3.16 × 10^6^ particles/μL	Serum (*n* = 6)	2023 [207]
Exosome	Ovarian Cancer	AuNPs	Antibody	SERS Spectroscopy	Buffer	1.5 × 10^5^ particles	N/A	–	2023 [208]
Exosome	Breast Cancer	Au@SiO_2_ NPs	PD-L1-Binding Peptide	SPR Spectroscopy	Buffer	0.16 particles/mL	10 × 10^3^ − 5 × 10^3^ particles/mL	Serum (*n* = 11)	2023 [209]
Exosome	Breast Cancer	Au@AgNPs and GO	Aptamer	SERS Spectroscopy	Buffer	1.5 × 10^2^ particles/mL	2.7 × 10^2^ − 2.7 × 10^8^ particles/mL	Serum (*n* = 11)	2023 [210]
Exosome	Prostate Cancer	Cu_2_O–CuO@Ag Nanowire	Antibody	SERS Spectroscopy	Buffer	89 particles/mL	2.79 × 10^2^ − 2.79 × 10^10^ particles/mL	Serum (*n* = 5)	2023 [211]

^a^The healthy donors’ biofluids, which are utilized to make model samples by spiking known concentrations of target analytes (e.g., recovery tests), are excluded here. To avoid confusion, we added only the biofluids obtained from actual patients (i.e., unknown samples) as “clinical samples” in this table

The early studies focused on the accurate quantification with high sensitivity of cancer-derived exosomes. Xia et al. demonstrated the colorimetric exosome detection method using CD63-specific aptamer-capped SWCNTs [197]. Since SWCNTs have peroxide-like activity, they can catalyze H_2_O_2_-mediated oxidation of TMB. This reaction was reduced by the addition of exosomes, which are expressed CD63 on the surface; thus, the amount of TMB oxidation is reduced and can be confirmed by the naked eye. The LOD of the sensor was 5.2 × 10^5^ particles/μL with a linear range between 1.84 × 10^6^ and 2.21 × 10^7^ particles/μL. The authors also found that approximately 1.5-fold more exosomes were found in the patients’ samples. Thakur et al. reported the LSPR biosensing method based on Au nanoislands (AuNIs) [196]. In their sensor design, randomly distributed nanostructures like AuNIs provide a convenient way to fabricate mass-producible and low-cost substrates for biosensors. By using an LSPR interferometer, the authors distinguished exosomes from other background vesicles. The LoD of the sensor was 0.194 μg/mL, and the linear range was in the range from 0.194 to 100 µg/mL. In the meantime, Zong et al. presented a SERS-based sandwich immunoassay method using a combination of magnetic nanobead (MB@SiO_2_) and silica-coated Au@Ag nanorod (Au@Ag NR@SiO_2_). Because the resulting signal is dependent on the amount of immunocomplex, the amount of the exosome in the sample can be measured qualitatively and quantitatively. The LOD of the system was 1200 exosomes with the detection ability of up to 10^5^ exosomes.

Second, the evaluation of multiple surface markers, which might be shared from their parental cells, is one of the important approaches. In this context, SERS-based detection also offers new perspectives in exosome profiling considering the complex and ambiguous nature of exosomes. Wang et al. proposed SERS-based detection methods for the screening of multiple exosomes simultaneously. The magnetic beads with gold shells (160 nm) contribute as a SERS substrate, while gold nanoparticles (17 nm) are utilized as SERS nanoprobes. The sandwich assay was conducted using three different aptamers and Raman reporters. The authors demonstrated the system with the exosomes that derived from three different cancer types (SKBR3, T84, and LNCaP cancer cells for breast, colorectal, and prostate cancer), and the LOD of the system was 32, 73, and 203 particles per microliter, respectively.

Zhang et al. Developed a simultaneous detection method for exosomal proteins using AuNPs and UCNPs. In this core-satellite design of probes, AuNPs served as a core, and three different types of UCNPs (yttrium, europium, and terbium) were arranged as satellites through three types of different aptamers (CD63, HER2, and EpCAM). Because the UCNPs were released when the aptamer recognized the specific marker on the exosomes, the authors collected and analyzed the detached UCNPs using ICP-MS and profiled the marker expression level. Zhang et al. utilized bimetallic nanoparticles and graphene oxide to construct both SERS nanoprobe and SERS substrates [210]. In this design, GO on the SERS substrate contributes to the enhanced surface area and the improved functionality of the receptor (V-shaped double-stranded DNA). The exosomes recreated from MCF-7 cells were analyzed with LOD down to 1.5 × 10^2^ particles/mL without any amplification strategy. Finally, the proposed system was validated using clinical samples and proved the ability to distinguish breast cancer patients, pancreatic cancer patients, and healthy individuals.

### Circulating tumor cells

Circulating tumor cells (CTCs) are rare cells that have been shed from the primary tumor to the bloodstream. Its frequency is usually in the range between 0 and 10 cells per millimeter of blood obtained from cancer patients [212]. It is extremely low level compared to red blood cells (RBCs, ~ 1 × 10^9^ per milliliter) and white blood cells (WBCs, ~ 5 × 10^6^ per milliliter). Although the presence of CTCs was first documented more than 150 years ago, their clinical utility was not validated until the late 1990s [213]. In 2004, the first CTC isolation method, called the CellSearch® system, had cleared by the US Food and Drug Administration (FDA). Allard et al. conducted large-scale clinical tests using this FDA-cleared system based on magnetic bead separation. With 2183 blood samples from 964 metastatic cancer patients having eight types of cancer, they found 0 to 23,618 CTCs per 7.5 mL of the blood. In contrast, the healthy individuals and patients having non-malignant disease did not have more than 2 CTCs, except for one case among 344 cases [43]. These early reports stimulated the CTC research to isolate the cancer cells from the whole blood. However, the advances had slowed at a certain point and caused troubles in verifying the clinical utility. There are several reasons that make CTC research challenging. Not to mention that the emergence of these cells is a very rare event, there has been speculation that the marker expression of CTCs usually changes during the detachment process [214, 215]. To address these issues, numerous studies have suggested various isolation and enrichment methods, including immunoaffinity-based methods and size-based methods [214, 216–218].

It is worth noting that CTCs are the most significant LB biomarkers because they are detached parts of the tumor, thus representing its origin. For these reasons, the detection of CTCs is somewhat different from the above-mentioned LB biomarkers. A comprehensive analysis should be accompanied by sensitive detection to detect down to a few cells. Because CTCs are whole packages containing proteins and nucleic acids, avoiding cell rupture and gently retrieving of viable CTCs for downstream analysis are also important. In addition, the heterogeneity of CTCs and a lack of their specific surface marker should be considered in the development of sensing technologies [219]. Notably, CTCs are the largest biomarker in liquid biopsy. The size of the cancer cells is usually above 10 μm in diameter, so arithmetically, it is approximately 10^3^ to 10^4^ times bigger than usual NPs. It means that multiple NPs can encode a single target cell, enabling signal accumulation [220]. Therefore, the distribution of multiple NPs on a cell may be equivalent to the mapping of cell surface marker expressions [2].

For the optical detection of CTCs, various detection methods using optical nanomaterials have been introduced. The representative studies are described in Table 9. Despite technical challenges, setting a guideline for CTC detection is relatively simple compared to ctDNAs and exosomes. The eventual performance needs to be reached for single-cell detection. Although the performance tends to vary by the setting of experimental conditions, several studies reported that the lowest LOD is close to a single-cell level in whole blood samples. Ruan et al. developed a SERS-based CTC detection system using nanoprobes consisting of triangular Ag nanoprisms and magnetic NPs. By a combination of FA-based isolation, magnetic enrichment, and SERS-based detection, the LOD of the system reached one CTC per mL. Afterward, the authors designed SERS-active magnetic NPs consisting of superparamagnetic iron oxide NPs with outer-arranged AuNPs. The LOD of CTC detection also reached 1 cell for mL. Wu et al. proposed a SERS-based method for the detection of CTCs in the blood. The authors prepare the Raman probes by encoding 4-mercaptobenzoic acid (4-MBA), followed by the functionalization with reductive bovine serum albumin (BSA) and folic acid (FA). They reported the LOD of 5 cells/mL with a linear range of 5 to 500 cells/mL.

**Table 9:** The optical nanomaterial-based biosensors for the detection of circulating tumor cells (CTCs)

Biomarker	Disease	Optical nanomaterial	Biorecognition element	Detection method	Matrix	Limit of detection	Linear range	Clinical sample [a]	Note
CTC	Lung Cancer Breast Cancer	AuNPs	EGF Ligand	SERS Spectroscopy	Blood	5 cells/mL	5–50 cells/mL	Blood (*n* = 20)	2011 [221]
CTC	Ovarian Cancer	Bismuth NPs (+ Magnetic NPs)	Folic Acid Ligands	X-ray Fluorescence Spectrometry	Buffer	~ 100 cells/mL	100–100,000 cells/mL	–	2012 [222]
CTC	Cancer	AuNPs	Antibody	Colorimetric Detection	Buffer	40 cells/mL	100–10,000 cells/mL	–	2014 [223]
CTC	Breast Cancer	AuNPs	Aptamer	Laser Desorption Ionization Mass Spectrometry (LD-IMS)	Diluted Blood	10 cells/mL	10–1000 cells/mL	–	2015 [224]
CTC	Lung Cancer	Magnetic UCNPs and Silicon NWs	Antibody	ULISA	Buffer	N/A	N/A	Blood (*n* = 21)	2015 [14]
CTC	Breast Cancer	AuNPs	Folic Acid Ligands	SERS Spectroscopy	Rabbit Blood	5 cells/mL	5–500 cells/mL	–	2015 [225]
CTC	Breast Cancer	CNDs, GQD, (+ Magnetic NPs)	Antibody	Fluorescence Detection	Blood	10 cells/mL	N/A	–	2016 [226]
CTC	Breast Cancer	Ag@Au Core–Shell NPs	Aptamer	Circular Dichroism (CD) Spectrometry	Blood	10 ± 6 cells/mL	50–10^5^ Cells/mL	–	2016 [227]
CTC	Breast Cancer	Au@Ag-Au Core–Shell NRs	Aptamer	SERS Spectroscopy	Blood	20 cells/mL	200–12,000 cells/mL	–	2017 [228]
CTC	Breast Cancer Ovarian Cancer	Triangular Ag Nanoprism (+ Magnetic NPs)	Folic Acid Ligands	SERS Spectroscopy	Blood	1 cells/mL	1–100 cells/mL	–	2018 [229]
CTC	Breast Cancer Ovarian Cancer	Fe_3_O_4_@nSiO_2_@mSiO_2_ NPs	Aptamer	Fluorescence Detection	Buffer	100 cells/mL	10^2^–10^5^ Cells/mL	–	2018 [230]
CTC	Breast Cancer	AuPd NPs	Aptamer	ECL Detection	N/A	40 cells/mL	10^2^–10^7^ Cells/mL	–	2018 [231]
CTC	Liver Cancer	Fe_3_O_4_@AgNPs	Antibody	SERS Spectroscopy	Blood	1 cells/mL	1–100 cells/mL	Blood (*n* = 18)	2018 [232]
CTC	Cancer	QDs (+ Magnetic NPs)	Antibody	Fluorescence Detection	Buffer	N/A	N/A	Blood (*n* = 9)	2019 [233]
CTC	Breast Cancer	UCNPs	Antibody	Time-Resolved Photoluminescence (TRPL) Spectroscopy	Buffer	1 cells/well	2–1024 cells/200 uL	Blood (*n* = 15)	2019 [234]
CTC	Breast Cancer	SPION-PEI@AuNPs (+ Magnetic NPs)	Aptamer	SERS Spectroscopy	Blood	1 cells/mL	1–25 cells/mL	Blood (*n* = 2)	2019 [235]
CTC	Breast Cancer	Au@CNDs	Aptamer	ECL Detection	N/A	34 cells/mL	100–10,000 cells/mL	–	2020 [236]
CTC	Breast Cancer	AuNPs	Aptamer	Fiber-Optic SPR	Buffer	49 cells/mL	N/A	–	2020 [237]
CTC	Breast Cancer	AuNPs	Antibody and Folic Acid Ligands	SPR Spectroscopy	N/A	1 cells/mL	10^1^–10^5^ cell/mL	–	2020 [238]
CTC	Cancer	Au Nanostar and Au Nanoflower	Aptamer	SERS Spectroscopy	N/A	5 cells/mL	5–200 cells/mL	–	2021 [239]
				Fluorescence Detection	N/A	10 cells/mL	10–200 cells/mL	–	
CTC	Breast Cancer	Black TiO_2_ NPs	Folic Acid Ligands	SERS Spectroscopy	Rabbit Blood	2 cells/mL	N/A	Blood (*n* = 6)	2022 [240]
CTC	Cancer	AuNPs	Aptamer	Fluorescence Detection	N/A	2 cells/200μL	10–100 cells/200μL	–	2023 [241]
CTC	Cancer	Au Nanostar@SiO_2_	Antibody	SERS Spectroscopy	Buffer	N/A	N/A	–	2023 [242]
CTC	Breast Cancer	Ag Nanorods (+ Magnetic NPs)	Aptamer	SERS Spectroscopy	Buffer	2 cells/mL	5–1000 cells/mL	–	2023 [243]

^a^The healthy donors’ biofluids, which are utilized to make model samples by spiking known concentrations of target analytes (e.g., recovery tests), are excluded here. To avoid confusion, we added only the biofluids obtained from actual patients (i.e., unknown samples) as “clinical samples” in this table

The evaluation of surface markers is a key to addressing heterogeneity issues of CTCs. Lin et al. designed 3-dimensional amorphous nitrogen-doped carbon nanocages as an SERS nanoprobe to image triple-negative breast cancer (TNBC) cells [220]. The identification of TNBC cells is important because they do not express the representative surface expression of breast cancer cells, such as estrogen receptor (ER), progesterone receptor (PR), and human epidermal growth factor receptor 2 (HER-2). It means that TNBC subtypes are not effectively treated by HER-2-targeted therapy. The authors quantitatively identified two types of TNBC cells, HCC 1806 and MDA-MB-231.

## Opportunities and future works

### Selection of nanomaterials

The attractiveness of nanomaterials originates from the unique features that are different from their bulk corresponding materials. In the nanometric dimension, the property of the materials drastically changes in every aspect, such as optical, electrical, and mechanical characteristics [13]. In addition, nanomaterials have significant surface-to-area ratios, thus enhancing the efficiency of the reaction. Various nanomaterials discussed above can be utilized as a sensitive optical nanoprobe solely or cooperatively via versatile detection strategies. The factors that can be considered in designing optical probes are size, shape, morphology, arrangement, structure, composition, physical/chemical characteristics, and compatibility with incorporated materials. As mentioned above, more studies have exploited two or more kinds of nanomaterials to induce a synergetic effect. In addition, the selection of nanoparticles is closely linked to the selection of appropriate detection systems.

Each nanomaterial has its own advantages and disadvantages, and these differences need to be considered during the selection of nanomaterials and/or the design of optical probes. Inorganic nanomaterials usually play a key role in optical probes due to their excellent optical properties. Metallic NPs like Au and Ag display excellent optical properties strongly dominated by the collective oscillation of free electrons on the metal surface; thus, their localized surface plasmon resonance (LSPR) can be tuned by size, shape, morphology, and interparticle distance [244]. However, individual metallic NPs are often not satisfactory for detecting ultra-low amounts of target analytes. Meanwhile, semiconductor nanomaterials like QDs show discrete electronic states through the “quantum confinement effect”, having broad absorption and narrow and symmetric emission bands with high photostability [245]. However, their applications are often restricted by probe size, toxicity, blinking effect, and difficulties in bioconjugation. Similar to QDs, carbon-based fluorescence nanodots can display size-tunable photoluminescence behavior through surface passivation with organic molecules. These biocompatible and chemically inert carbon NPs have recently gained much attention as a sensing probe for optical detection owing to favorable properties, such as low toxicity and non-blinking effect [246]. Unlike other carbon-based materials, however, these carbon NPs still have not established systematic and scalable protocols. UCNPs are an emerging class of optical nanomaterials based on upconversion luminescence, an interesting phenomenon defined as the conversion of long-wavelength radiation to short-wavelength radiation (e.g., from infrared or near-infrared (NIR) to the visible range) [247, 248]. So, they are useful in biosensing and bioimaging due to the low autofluorescence background, low photobleaching, as well as narrow emission bandwidth. However, several challenges remain due to their complicated synthesis methods, causing trade-offs between toxicity and efficiency [249].

Sometimes, these nanomaterials themselves are not suitable for liquid biopsy applications. First of all, most nanomaterials are hard to control in complex media because of their inherently sensitive nature to ionic substances. It implies the difficulties in exploiting them under physiological conditions or real clinical samples, decolorizing the distinct merit of optical detection. Furthermore, some nanomaterials are not fully evaluated in terms of their biocompatibility. For these reasons, the hybridization of one or more nanomaterials by constructing nanoarchitectures can also be a rational solution. Nanoarchitectures can be designed in the aspect of composition (silica and polymeric materials), structure (core–shell and yolk-shell), or function (magnetic and catalytic reaction). Magnetic NPs or microbeads not only have a long history in biomedical applications as preferred solid support but are also one of the first successful strategies in liquid biopsy because of signal enrichment and effortless purification. Silica NPs have been considered an ideal matrix for phosphors or small metal crystals because they are transparent to light [250]. In addition, silicon NPs have several advantages, including chemical/physical stabilities and hydrophilic surfaces, allowing easy modification [251]. The silica encapsulation offers increased detectability compared to the individual phosphors or small metal crystals and expands the usability with size controllability and multifunctional properties based on the water solubility [252–254]. Polymeric NPs are also an ideal candidate due to their soft and biocompatible nature. More importantly, polymeric material has the ability to react against the environment (e.g., stimuli-responsive behavior); so, their adoption can provide additional dynamic functionality to the probes [255].

**Fig. 2:**
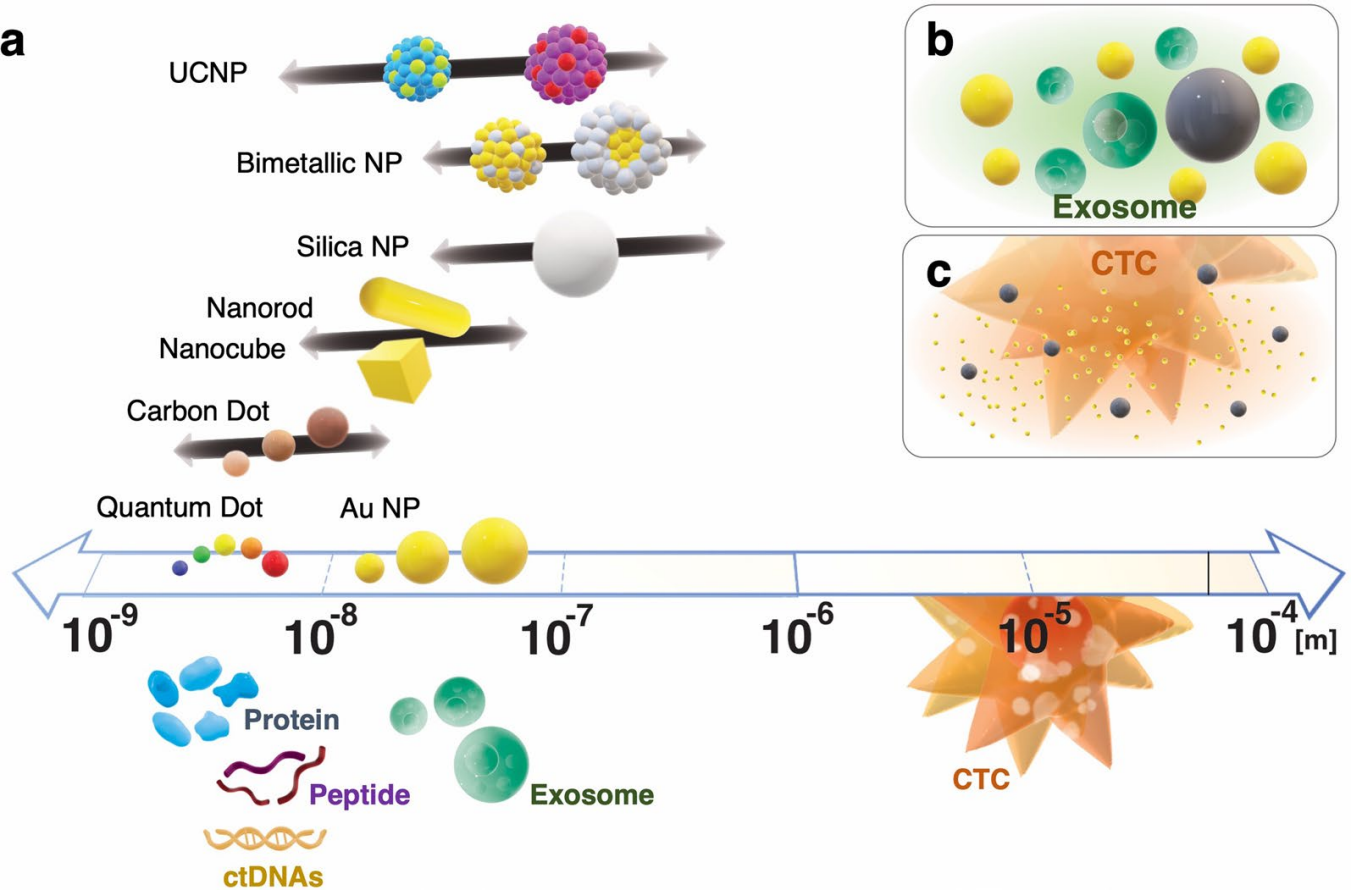
The representative optical nanomaterials and LB biomarkers. **a** the comparison of nanomaterials and LB biomarkers in size with scale; **b** illustration of AuNP-based detection of exosomes with the help of magnetic NPs; **c** illustration of AuNP-based detection of CTCs with the help of magnetic NPs

The combination of the optical nanomaterials to detect LB biomarkers is a fascinating point in this subject. The LB biomarkers are very different from each other in characteristics and exist in different metric regimes from nanoscale to microscale. In Fig. 2, we displayed their degree of relativity with the illustration. The largest LB biomarkers are larger than 10 µm (e.g., CTCs), whereas the smallest LB biomarkers are smaller than 10 nm (e.g., microRNA). It offers interesting points in the selection of proper optical NPs and their size. Most protein and LB biomarkers are comparable to QDs and slightly smaller than metallic NPs (less than one order). Exosomes (30 to 150 nm) have a wide size distribution range comparable to various nanomaterials. (Fig. 2b). On the other hand, CTCs are much bigger than the largest nanoparticles (~ 150 nm), with almost two orders of differences (Fig. 2c). Therefore, smaller optical NPs detect CTCs by one-to-many correspondence. It is sort of analogous to the relation between protein and cell via encoding the surface of the cells with NPs; thus, it can provide information regarding the heterogeneity of the CTCs [256].

### Assignment for clinical applications

Liquid biopsy is now entering the plateau phase. Despite much anticipation, there still are many hurdles to overcome until it is established as a part of standard procedure for clinical decisions. The challenges stem from both the conceptual difficulties in liquid biopsy and the exceptional characteristics of each LB marker. Although we cover both traditional biomarkers and newly emerging LB biomarkers together under the broad concept of liquid biopsy, there will be slight differences in the future approaches. In the case of the traditional biomarkers (e.g., protein and peptide), enhanced sensitivity and accurate quantification are the primary objectives. On the other hand, newborn LB biomarkers (i.e., CTC, exosome, ctDNA) inherently possess more complicated problems, such as rarity, heterogeneity, and short half-life, compared to amino acid-based short chains or macromolecules. Aside from the sensing performance, an effective enrichment process should also be considered for these special classes of LB biomarkers. For example, the adoption of magnetic nanobeads or microbeads is a representative example. Since the first FDA-cleared CTC detection assay was introduced in 2007, magnetic separation occupies a large portion of liquid biopsy technology, and it is very suitable for optical detection using various NPs. Numerous studies and product prototypes utilize magnetic beads as a sort of substrate of the immunoreaction to be designated by optical NPs, thus achieving highly sensitive detection from the enriched samples.

In terms of clinical utility, traditional biomarkers have two different sides of points. They have been utilized in the diagnosis for a long time, whereas their already-proven limitation is obvious. The enhancement of analytical sensitivity and specificity might be helpful in the early screening of the disease. However, these biomarkers cannot provide in-depth information for treatment as well as prognosis. On the other hand, newborn LB biomarkers have potentials that have never been unveiled, even though their feasibility has not been fully proven due to the investigation with small cohorts and/or results in disagreement. As we mentioned above, the major reason for these conflicted results is a lack of a standard sample preparation method [255]. Currently, there is no consentaneous sampling, handling, or storage method in liquid biopsy. Likewise, one critical problem in biosensing development is a lack of reproducibility [257]. Although the studies discussed in this review report excellent sensing performance with additional functionalities to solve the current limitations in liquid biopsy, there will be a gap between lab-scale testing and practical applications. In conclusion, the actual clinical utility of LB biomarkers could be evaluated when accurate and reproducible methods are established with an efficient enrichment.

In the meantime, combined analysis would be a reasonable approach to overcoming the current obstacles in liquid biopsy. Two or more LB biomarkers provide complementary information about the disease. In addition, the drawback of each LB biomarker can sometimes be covered by other LB biomarkers. Traditional biomarkers have always been a starting point to validate the clinical meaning of ctDNA. Cohen et al. conducted a test to compare KRAS mutation in both ctDNA and protein LB biomarkers (AFP, CA15-3, CEA, CA-125, etc.) from 221 pancreatic cancer patients and 182 control patients [258]. Rossi et al. conducted a combinational analysis of CTCs and cfDNAs of metastatic breast cancer patients using CellSearch® systems and Guardant360, respectively [259]. Similarly, Ye et al. analyzed the samples obtained from metastatic breast cancer patients using CellSearch® systems and RT-PCR, respectively [260]. The role of exosomes as the only abundant LB biomarkers has also been investigated. Kim et al. designed a hydrogel-based immunoassay to isolate both CTCs and exosomes from colorectal cancer patients’ blood samples [10]. Although there was no clear evidence of the correlation between CTCs and exosomes in terms of concentration, the degradable hydrogel-based effortless collection of two different LB biomarkers from the identical sample provides an opportunity to contribute to further analysis.

New diagnostic technologies should satisfy diagnostic accuracy requirements for utilization in hospital routines. There are many criteria for adopting new diagnostic technologies in clinical practice. Guatt et al. categorized these requirements, including technological capability, range of possible use, diagnostic accuracy, impact of healthcare providers, therapeutic impact, and patient outcome [261]. In addition, they need to be technologically and psychologically accepted by physicists, biochemists, physiologists, and other healthcare providers.

## Conclusion

In this review, we summarized the recent advances in liquid biopsy using optical nanomaterials, such as metallic NPs, QDs, UCNPs, and carbon nanomaterials. Optical detection, one important branch in biosensor history, possesses a simple and straightforward nature with less disturbance to environmental factors, thus well-fitting to a biofluid-based setting of liquid biopsy. Furthermore, the tailored design of each nanoprobe achieves signal enhancement and also widens the dynamic range. The advances in sensing performance will accelerate further studies from molecular biology to medicine and may contribute to the understanding of the veiled characteristics of LB markers. In spite of the above-mentioned problems, we expect the attention to liquid biopsy to be continued owing to the significance of minimally invasive diagnostic methods. Also, the concept itself will keep refining, expanding, and even evolving from one of the topics in oncology to a significant issue in the entire field of medicine and public health. The early diagnosis of disease with convenient and frequent medical check-ups would enormously lower the socioeconomic burden of disease. Eventually, these efforts enable us to develop the liquid biopsy assay in a real-world clinical setting.

## Data Availability

Not applicable.

## Abbreviations

AFP: Alphafetoprotein
BTA: Bladder tumor antigen
BNP: Brain natriuretic peptide
CD: Circular dichroism
CEA: Carcinoembryonic antigen
CND: Carbon nanodot
cfDNA: Circulating free DNA
ctDNA: Circulating tumor DNA
CL: Chemiluminescence
CQD: Carbon quantum dot
CTC: Circulating tumor cell
CYFRA: Cytokeratin fragment
ECL: Electrochemiluminescence
ELISA: Enzyme-linked immunosorbent assay
FRET: Fluorescence resonance energy transfer
GQD: Graphene quantum dot
HBsAg: Hepatitis B surface antigen
HCcAg: Hepatitis C core antigen
ICP-MS: Inductively coupled plasma-mass spectrometry
LB: Liquid biopsy
LD-IMS: Laser desorption/ionization mass spectrometry
LFA: Lateral flow assay
LRET: Luminescence resonance energy transfer
LSPR: Localized surface plasmon resonance
MEF: Metal enhanced fluorescence
NGS: Next generation sequencing
NIR: Near-infrared
NMP: Nuclear matrix protein
NP: Nanoparticle
NR: Nanorod
NSE: Neuron-specific enolase
NTproNP: N-terminal proBNP
PNA: Peptide nucleic acid
PSA: Prostate cancer antigen
QD: Quantum dot
SERS: Surface enhanced raman scattering
SPR: Surface plasmon resonance
THMS: Triple-helix molecular switch
TPRL: Time-resolved photoluminescence
UCNP: Upconverting nanoparticles
ULISA: Upconversion-linked immunosorbent assay
UV: Ultraviolet
XFS: X-ray fluorescence spectroscopy
